# Engrafted NSG-SGM3 humanized mice spontaneously produce human immunoglobulins including IgE

**DOI:** 10.3389/fimmu.2025.1628194

**Published:** 2025-08-25

**Authors:** Erica V. Lin, Rebecca A. Krier-Burris, Kristina A. Sokol, Betania Arce, Natalia M. Vilela, Robert G. Hamilton, Bruce S. Bochner, Melanie C. Dispenza

**Affiliations:** ^1^ Division of Allergy and Clinical Immunology, Department of Medicine, Johns Hopkins University School of Medicine, Baltimore, MD, United States; ^2^ Division of Allergy and Immunology, Department of Medicine, Northwestern University Feinberg School of Medicine, Chicago, IL, United States

**Keywords:** anaphylaxis, B cell, humanized mice, IgE, NSG-SGM3

## Abstract

NSG-SGM3 humanized mouse models are well-suited for studying human immune physiology but are technically challenging and expensive. We previously characterized a simplified NSG-SGM3 mouse, engrafted with human donor CD34^+^ hematopoietic stem cells without receiving prior bone marrow ablation or human secondary lymphoid tissue implantation, that still retains human mast cell- and basophil-dependent passive anaphylaxis responses. Its capacities for human antibody production and human B cell maturation, however, remain unknown. Here, we show that NSG-SGM3 mice engrafted without prior marrow ablation spontaneously produce all human antibodies, including IgE, without deliberate sensitization. These human IgE antibodies are polyclonal with unexpected specificities to diverse allergens, such as millet, egg, and wasp venom, that are otherwise absent from the mouse diet or housing environments. Furthermore, human CD138^+^ CD27^+^ plasma cell and CD20^+^ CD27^+^ memory B cell populations can be expanded from naïve engrafted NSG-SGM3 splenocytes in response to human CD40L and IL-4 cytokine stimulation *ex vivo*. Engrafted NSG-SGM3 mice, but not non-engrafted controls, also exhibit dose-dependent passive systemic anaphylaxis responses when challenged with goat anti-human IgE. In contrast, no anaphylaxis responses were observed in humanized NSG-SGM3 mice challenged with select food allergens. Together, our results demonstrate that engrafted NSG-SGM3 mice without prior ablation spontaneously produce abundant functional human antibodies, including polyclonal IgE that can facilitate anaphylaxis. These mice also unexpectedly possess the upstream capacity to support human B cell maturation into antibody-producing plasma cells and memory B cells. Our simpler humanized NSG-SGM3 model therefore reveals novel insights into dynamics of human B cell maturation, homing, and differentiation that facilitate the generation of a basal, functional, polyclonal IgE repertoire without deliberate sensitization.

## 1 Introduction

Humanized mouse models are powerful tools for studying aspects of human immune physiology not fully reconstituted by the native murine immune system (1–3), such as allergy and anaphylaxis. A common humanized mouse model is the nonobese diabetic severe combined immunodeficiency common gamma chain-deficient (NOD *scid* gamma; NSG) mouse. NSG mice are immunodeficient and thus suitable for humanization due to mutations in IL-2R common gamma chain to deplete natural killer cells and *Prkdc* (*scid*) to deplete B and T cells (2–4). Traditional NSG models involve a three-pronged approach for humanization: (1) irradiation or chemical ablation of murine host bone marrow, (2) surgical implantation of human fetal liver and/or thymic tissue, and (3) engraftment with human CD34^+^ hematopoietic stem cells (HSCs) (5–8). This older approach to creating “bone marrow, liver, thymus” (BLT) mice assumes that the original murine hematopoietic compartment may interfere with human HSC engraftment, and that endogenous murine lymphoid tissues are not sufficient milieus to support functional human HSC maturation without additional human lymphoid implants.

The next-generation NSG-SGM3 (NOD *scid*-IL2Rg^null^-3/GM/SF) strain additionally expresses human transgenes for SCF, GM-CSF, and IL-3, cytokines which support the survival, proliferation, and maturation of human hematopoietic-derived granulocytes, including mast cells and basophils, even without implantation of human lymphoid tissue (4, 9–11). As a result, both BLT NSG-SGM3 mice as well as NSG-SGM3 mice engrafted after prior irradiation but lacking lymphoid explants can undergo passive systemic anaphylaxis (PSA) that is driven by human mast cells and basophils in response to allergen challenge (5, 12, 13). NSG-SGM3 mice are thus well-suited for modeling granulocyte- and IgE-dependent allergic responses and anaphylaxis in humans (9, 11, 12). Creating these mice, however, is very time-consuming and expensive. Furthermore, NSG-SGM3 mice engrafted after irradiation are prone to developing lymphomas and hemophagocytic lymphohistiocytosis (HLH)-like and/or macrophage activation syndrome (MAS)-like pancytopenia with older age (14–17).

We previously characterized a newer and simplified model of humanized NSG-SGM3 mice which are engrafted without prior bone marrow ablation or human lymphoid tissue implantation. In our model, 3–4-week-old NSG-SGM3 mice receiving one intravenous injection of human cord blood HSCs without preconditioning were similarly capable of supporting robust human HSC engraftment, including mature human mast cells and basophils, by 16 weeks post-injection, while their residual murine CD45^+^ leukocytes dwindled over time (8). Notably, PSA responses for our simplified model were just as robust as for BLT mice (5, 8), suggesting that the physiology of human HSC maturation and downstream anaphylaxis remain intact despite present residual host murine immune cells. Furthermore, NSG-SGM3 mice without prior irradiation survive longer without HLH-like post-engraftment complications despite comparable HSC engraftment efficiency (14). These data put forth several advantages of our simplified model, especially in light of its intact granulocyte physiology.

It still remains unknown, however, whether NSG-SGM3 mice engrafted without prior marrow ablation can also support human antibody production and human B lymphocyte development. While NSG-SGM3 mice engrafted after irradiation and BLT NSG-SGM3 mice have been shown to make basal serum human IgM and IgG at older age (4, 18), these mice are again prone to severe immune dysregulation (15), with possibly lowly- or non-functional antibody. In addition, NSG-SGM3 mice engrafted without prior ablation or human tissue lymphoid implantation make peanut-specific IgE after oral gavage with peanut, which suggests some functional human plasma cell differentiation (11). It is therefore poorly understood whether human HSC-derived human B cells can produce functional human antibody in the setting of a non-ablated murine background in NSG-SGM3 mice, as well as why these human B cells can survive in host murine lymphoid milieus without human lymphoid tissue.

Here, we show for the first time that NSG-SGM3 humanized mice engrafted without prior ablation can produce spontaneous human antibodies of all isotypes without deliberate sensitization, including polyclonal and functional human IgE. Our work also characterizes the human antibody and cytokine landscapes and identifies putative Th2 cytokine-responsive B cell populations in our simplified NSG-SGM3 humanized mouse model, with an additional novel focus on functional profiling of human IgE.

## 2 Materials and methods

### 2.1 Mice

C57BL/6J wildtype (“WT”; “B6”; 000664) and NOD.Cg-*Prkdc^scid^ Il2rg^tm1Wjl^* Tg(CMV-*IL3,CSF2,KITLG*)1Eav mice (“NSG-SGM3”; 013062) were purchased from Jackson Laboratory. All mice were female sex and littermates unless otherwise stated and maintained in barrier housing in specific-pathogen free animal facilities on the Johns Hopkins Bayview Medical Center campus.

NSG-SGM3 mice were engrafted as previously described at 3–4 weeks old with a single retroorbital injection of 5 x 10^5^ human cord blood CD34^+^ cells (Lonza Poietics) resuspended in sterile PBS (Sigma) (8). Successful engraftment of human cells was confirmed 12 weeks after injection using flow cytometry analysis of whole blood stained with appropriate fluorescent antibodies and processed on a BD Accuri C6 cytometer (BD Biosciences) as previously described (8). Serum samples were collected via retroorbital bleed after 16 weeks of engraftment and at least two weeks prior to splenocyte culture.

NSG-SGM3 mice were fed autoclaved Teklad Global 18% Protein Extruded Rodent Diet (Inotiv Inc.) (**Supplementary Table S1**) and received enrofloxacin (Baytril; Elanco US Inc) in sterile drinking water as prophylaxis against opportunistic infections. WT mice were housed in normal rodent housing and treated with untreated filtered drinking water. WT and non-engrafted NSG-SGM3 mice were age-matched with 20–24-week-old engrafted NSG-SGM3 mice at the start of culture experiments. All animal protocols were conducted under the approval and regulation of the Institutional Animal Care and Use Committee at Johns Hopkins University School of Medicine.

### 2.2 Mouse whole blood and serum samples

For murine whole blood samples, whole blood was collected by retroorbital bleeds into purple-cap Microtainer Tubes containing EDTA (BD Biosciences) at room temperature. Samples were lysed twice with 1x multi-species RBC lysis buffer (Invitrogen) in 5 mL polystyrene round-bottom flow cytometry tubes (Corning) at room temperature in the dark and washed with 1x PBS before antibody staining for flow cytometry. For serum samples, murine whole blood was first collected by retroorbital bleeds into serum separator Microtainer Tubes (BD Biosciences). Tubes were allowed to clot at room temperature for at least 30 minutes and centrifuged at 1000 x g for 10 minutes to separate serum. Serum was stored at -20°C until analysis.

### 2.3 Quantitative antibody and cytokine measurements

The R-PLEX Human IgE assay (Meso Scale Discovery) and the T-PLEX Human Isotyping Panel 1 kit for human IgM, IgG, and IgA (Meso Scale Discovery) were used to detect levels of human antibodies in NSG-SGM3 mouse sera and in supernatants of *ex vivo* splenocyte cultures after 96 hours. Lyophilized wildtype mouse sera reconstituted in 1x PBS (Sigma) and purified mouse IgE (BD Biosciences) were used as controls to ensure lack of cross-reactivity of the human Meso Scale assays with murine antibodies. Human IgE levels from pooled samples of mouse sera were also confirmed using the ImmunoCAP total serum IgE assay (Thermo Fisher Scientific). Cytokine concentrations of NSG-SGM3 mouse sera were measured using R-PLEX kits for SCF, U-PLEX for GM-CSF and IL-3, U-PLEX for human IL-4 and murine IL-4, and a custom V-PLEX kit for human IL-4, IL-6, IL-13, IFN-γ, and TNF-α (all from Meso Scale Discovery). Recombinant human IL-4 (R&D Biosciences) and murine IL-4 (R&D Biosciences) were used as controls to confirm lack of interspecies cross-reactivity of the Meso Scale IL-4 assays. Sample preparation and processing were performed per manufacturer protocol. Processed plates were read on a MESO QuickPlex SQ 120 machine (Meso Scale Discovery).

### 2.4 Specific IgE profiling

Mouse sera were pooled into three samples from 5–6 mice each for analysis on the Allergy Xplorer 2 (ALEX^2^) (Macro Array Diagnostics), which quantifies specific IgE antibodies for 295 clinically relevant allergens (19, 20). In brief, samples were diluted 1:5 in an assay diluent containing MUXF, a carbohydrate cross-reactive determinant (CCD)-blocking agent (21). From this, 500 μL of sample was incubated on a chip cassette for two hours. The chip was then washed and incubated with substrate for eight minutes, after which the reaction was stopped. Results were read on the ImageExplorer instrument (Macro Array Diagnostics) and reported in kUA/L following interpolation by Raptor software from a built-in total IgE calibration curve. Levels >0.10 kUA/L were considered positive.

### 2.5 Splenocyte harvest and *ex vivo* cultures

To harvest and culture bulk *ex vivo* splenocytes, we modified published protocols originally designed for the harvest and *ex vivo* cultures of CD19^+^ B cells (22, 23). Briefly, spleens of WT, non-engrafted NSG-SGM3, or engrafted NSG-SGM3 mice were harvested and mashed through a 70 μm cell strainer (Corning Life Sciences) into a 6-well flat-bottom plate containing RPMI-1640 media (Corning) supplemented with 2.05 mM L-glutamine (DiagnoCine Precision), 15% fetal calf serum (Sigma Aldrich), 1% penicillin and streptomycin (Gibco), and 50 μM 2-mercaptoethanol (Sigma). Cells were spun at 500 x g for five minutes at room temperature. Erythrocytes were then lysed with 1x multi-species RBC lysis buffer (Invitrogen) and remaining bulk splenocytes were washed and spun.

Harvested bulk WT, non-engrafted NSG-SGM3, or engrafted NSG-SGM3 splenocytes were initially plated in 6-well flat-bottom plates at 1 x 10^6^ cells/mL in supplemented RPMI media as above, and incubated from 0 to 48 hours with either 1 μg/mL mouse anti-human CD40 monoclonal antibody (anti-hCD40; BD Pharmingen) and 10 ng/mL recombinant human IL-4 (hIL-4; R&D Systems) anti-human stimulatory cytokines, or 1 μg/mL hamster anti-mouse CD40 monoclonal antibody (anti-mCD40; BD Biosciences) and 10 ng/mL recombinant mouse IL-4 (mIL-4; R&D Systems) anti-mouse stimulatory cytokines. At 48 and 72 hours, cells were replated in 12-well flat-bottom plates at 1 x 10^6^ cells/mL in fresh supplemented RPMI media and re-incubated at both timepoints with the appropriate anti-human or anti-mouse stimulatory cytokines at 0.5 μg/mL CD40L and 5 ng/mL IL-4 concentrations. Culture supernatants were obtained at the 96-hour timepoint and stored at -20°C until further analysis using MSD Multiplexing kits as above (Meso Scale Discovery). Cultures were maintained in a 37°C incubator at 5% CO_2_ for 96 hours total before harvest.

### 2.6 Human donor whole blood samples

Human whole blood samples were obtained from healthy donors consented under a protocol approved by the Institutional Review Board at Johns Hopkins University School of Medicine. Human whole blood samples were collected via standard phlebotomy and drawn into purple-cap BD Microtainer blood collection tubes containing EDTA (BD Biosciences) prior to preparation. Samples were lysed with 1x multi-species RBC lysis buffer (Invitrogen) in 5 mL polystyrene round-bottom flow cytometry tubes (Corning) at room temperature in the dark and then washed with 1x PBS before proceeding with antibody staining for flow cytometry.

### 2.7 Flow cytometry and analysis of *ex vivo* cultures and whole blood samples

Antibody and miscellaneous reagent concentrations for flow cytometry were titrated based on manufacturer protocols (**Supplementary Table S2**).

For the preparation of *ex vivo* splenocyte cultures at 0 hour or 96 hour timepoints, harvested cells were washed with 1x PBS, assessed for viability using LIVE/Dead Fixable Aqua (Invitrogen), incubated with a blocking cocktail containing rat anti-mouse CD16/CD32 (mouse BD Fc block; BD Biosciences), human BD Fc Block (BD Biosciences), and True-Stain Monocyte Blocker (BioLegend), and subsequently stained with 100 μL of either anti-human or anti-mouse antibody cocktail (**Supplementary Table S2**). For preparation of human donor and mouse whole blood, RBC-lysed and washed cells were assessed for viability using LIVE/Dead Fixable Aqua, incubated with a blocking cocktail containing human BD Fc block and True-Stain Monocyte Blocker, and subsequently stained with 100 μL of either anti-human or anti-mouse antibody cocktail (**Supplementary Table S2**). All stained mouse and human samples were fixed in 50 μL 4% paraformaldehyde in 1x PBS and stored at 4°C until time of analysis. Fixed and stored samples were resuspended in 150 μL 1x PBS immediately prior to cytometer analysis. UltraComp eBeads (Invitrogen), ArC amine reactive beads (Invitrogen), and ArC amine negative beads (Invitrogen) were utilized per manufacturer protocol for compensation of the appropriate antibodies. Samples were run on a Cytek Aurora cytometer (Cytek Biosciences).

Flow cytometry samples were analyzed on FlowJo v10 software (Tree Star) using gating strategies specifically designed to exclude follicular, marginal zone, and germinal center B cells that may otherwise contribute to false positive signal from the final gate (**Supplementary Figures S1, S2**). Specifically, human PC were identified by gating sequentially on singlet, live, human CD138^+^ human CD19^var^, human IgD^-^, human CD23^-^, human CD138^+^ human CD27^+^ cells (**Supplementary Figure S1**). Human MBC were gated sequentially on singlet, live, human CD19^+^ human CD138^-^, human IgD^-^, human CD23^-^, human CD20^+^ human CD27^+/hi^ cells (**Supplementary Figure S1**). Mouse PC and PB were gated sequentially on singlet, live, mouse CD138^hi^, mouse IgD^-^ mouse CD22^-^, mouse/human GL-7^lo^, mouse/human B220^lo^ cells; thereafter, mouse PC were gated as mouse CD138^+^ mouse CD19^+^ cells, and mouse PB were gated as mouse CD138^+^ mouse CD19^-^ cells (**Supplementary Figure S2**). Mouse MBC were gated sequentially on singlet, live, mouse CD138^-^, mouse IgD^-^ mouse CD22^-^, mouse/human GL-7^lo^, mouse CD273^+^ mouse/human B220^+^ cells (**Supplementary Figure S2**). Minimal cross-reactivity between anti-human and anti-murine antibodies with murine and human samples, respectively, were validated using murine whole blood, murine splenocytes, and human donor whole blood (**Supplementary Figure S3**).

### 2.8 *In vivo* systemic anaphylaxis

Anaphylaxis experiments were performed at least 16 weeks post-engraftment. For direct targeting of IgE, engrafted and non-engrafted NSG-SGM3 mice were injected intraperitoneally with 100 ng or 1 µg of goat polyclonal anti-human IgE (Hybridoma Reagent Laboratory) or equivalent amounts of normal polyclonal goat IgG isotype control (R&D Systems) diluted in sterile 1x PBS. To assess anaphylaxis responses, core body temperature was measured using a rectal probe (Kent Scientific), and clinical score was assessed every ten minutes over the course of one hour. Clinical scores from zero to five were as follows: 0) asymptomatic; 1) scratching; 2) piloerection, facial edema, 3) labored breathing, reduced activity; 4) coma or unresponsiveness; and 5) death. In the event of death, temperature measurements for that specific mouse were terminated.

For planned food challenges, enough blood was obtained from two engrafted NSG-SGM3 mice to allow for full specific IgE profiling of their individual sera on the ALEX^2^ chip. While numerous low-level positives were noted, consistent with the previously described pooled samples, both mice had specific IgE antibodies to several foods, of which three foods were selected for challenges based on ease of administration via oral gavage and lack of prior exposure to these allergens. Mice were challenged by oral gavage with 200 μL nonfat cow’s milk (corresponding to 6.6 mg protein; Whole Foods), 20 mg yeast dissolved in water (11.3 mg protein; Anthony’s Brewer’s Yeast Powder), and 56 mg hazelnut paste diluted in water (8.3 mg protein; Pariani Unsweetened Hazelnut Paste) in separate experiments. Anaphylaxis responses were assessed as described above.

### 2.9 Basophil activation testing

Human blood samples were obtained from healthy volunteer donors after informed consent under an Institutional Review Board-approved protocol. Blood was collected via standard phlebotomy and drawn into 4 mL lithium heparin phlebotomy tubes (BD Biosciences). Whole blood was incubated for 30 minutes at 37°C with goat polyclonal anti-human IgE (Hybridoma Reagent Laboratory), polyclonal goat IgG isotype control (R&D Systems), or 1 µM N-formylmethionyl-leucyl-phenylalanine (Sigma), diluted in PAGCM buffer containing piperazine-N,N′-bis[2-ethanesulfonic acid], bovine serum albumin (MP Biomedicals), glucose (Sigma-Aldrich), 1.7 mM calcium (Sigma), and 1.7 mM magnesium (Sigma). After stimulation, cells were fixed with BD Phosflow Fix Buffer (BD Biosciences), centrifuged for five minutes at 400 x g, and resuspended in Pipes buffer containing 1 mM EDTA and 0.25% bovine serum albumin.

In preparation for analysis, cells were blocked with 1 mg/mL non-specific human IgG (MB Biological), incubated with fluorescently-conjugated monoclonal anti-human CD63 (1:1000 dilution; BD Pharmingen) and anti-human FcϵRIα (clone CRA-1, 1:250, Life Technologies) antibodies at room temperature for 25 minutes, and then stained with secondary antibodies anti-CD123-PE (1:100, BD Biosciences), anti-mouse IgG2b-AlexaFluor488 (1:1000, Life Technologies), and anti-mouse IgG1-AlexaFluor647 (1:1000, Life Technologies) at room temperature for 25 minutes. Samples were analyzed on a BD Accuri C6 flow cytometer (BD Biosciences) using a previously-defined gating strategy (24). The percentage of CD63^+^ basophils was recorded for each sample.

### 2.10 Quantification and statistical analysis

**Figure 1:**
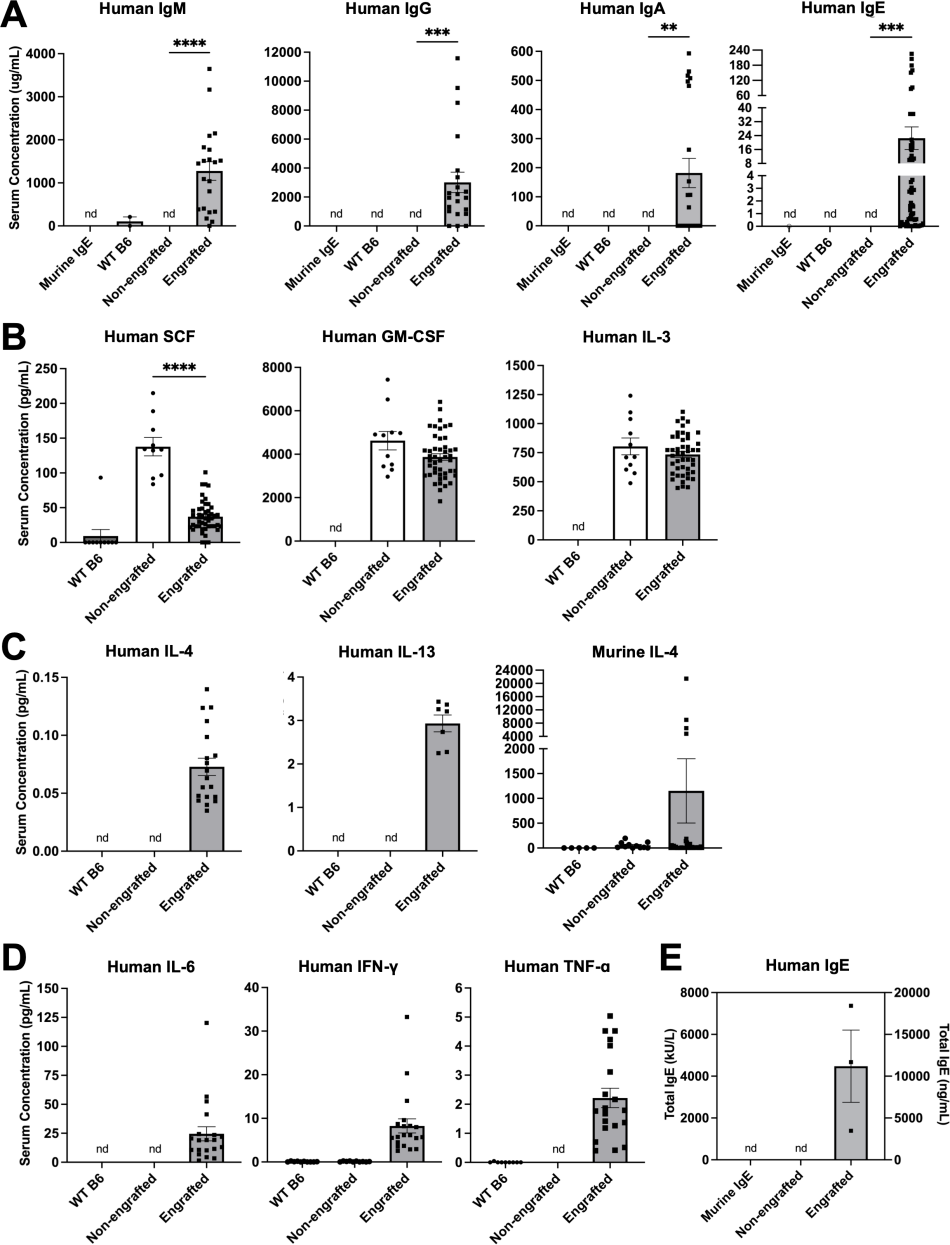
Engrafted NSG-SGM3 mice spontaneously produce abundant circulating human antibodies, including IgE. **(A)** Serum concentrations of human IgM, IgG, IgA, and IgE antibodies for WT, non-engrafted NSG-SGM3, and engrafted NSG-SGM3 mice. Murine anti-DNP IgE antibody was used as an isotype control (nd = not detectable; *n* = 2 WT, 3 non-engrafted NSG-SGM3, and 22 engrafted NSG-SGM3 mice compiled from two different experiments for IgM, IgG, and IgA; *n* = 14 WT, 7 non-engrafted NSG-SGM3, and 63 engrafted NSG-SGM3 mice compiled from three different experiments for IgE). **(B)** Serum concentrations of human SCF, GM-CSF, and IL-3 cytokines for WT (*n* = 10–11), non-engrafted NSG-SGM3 (*n* = 10–11), and engrafted NSG-SGM3 (*n* = 46) mice. **(C)** Serum concentrations of human IL-4, human IL-13, and murine IL-4 Th2 cytokines for WT (*n* = 5–10), non-engrafted NSG-SGM3 (*n* = 10–11), and engrafted NSG-SGM3 (*n* = 11–37) mice. **(D)** Serum concentrations of human IL-6, IFN-γ, and TNF-α Th1 cytokines for WT (*n* = 10), non-engrafted NSG-SGM3 (*n* = 10–11), and engrafted NSG-SGM3 (*n* = 20) mice. **(E)** Total serum human IgE levels in engrafted versus non-engrafted NSG-SGM3 mice, determined by secondary ImmunoCAP assay from ALEX^2^ allergen chip analysis. Murine IgE antibody was used as an isotype control. Values reflect serum concentrations in kU/L (left y-axis) or ng/mL (right y-axis) (*n* = 3 samples pooled from five mice each). Error bars in **(A–E)** represent SEM. P values < 0.05 were considered significant (**p < 0.01; ***p < 0.001; ****p < 0.0001). Welch’s *t*-test was used for **(A, B)**.

**Figure 2:**
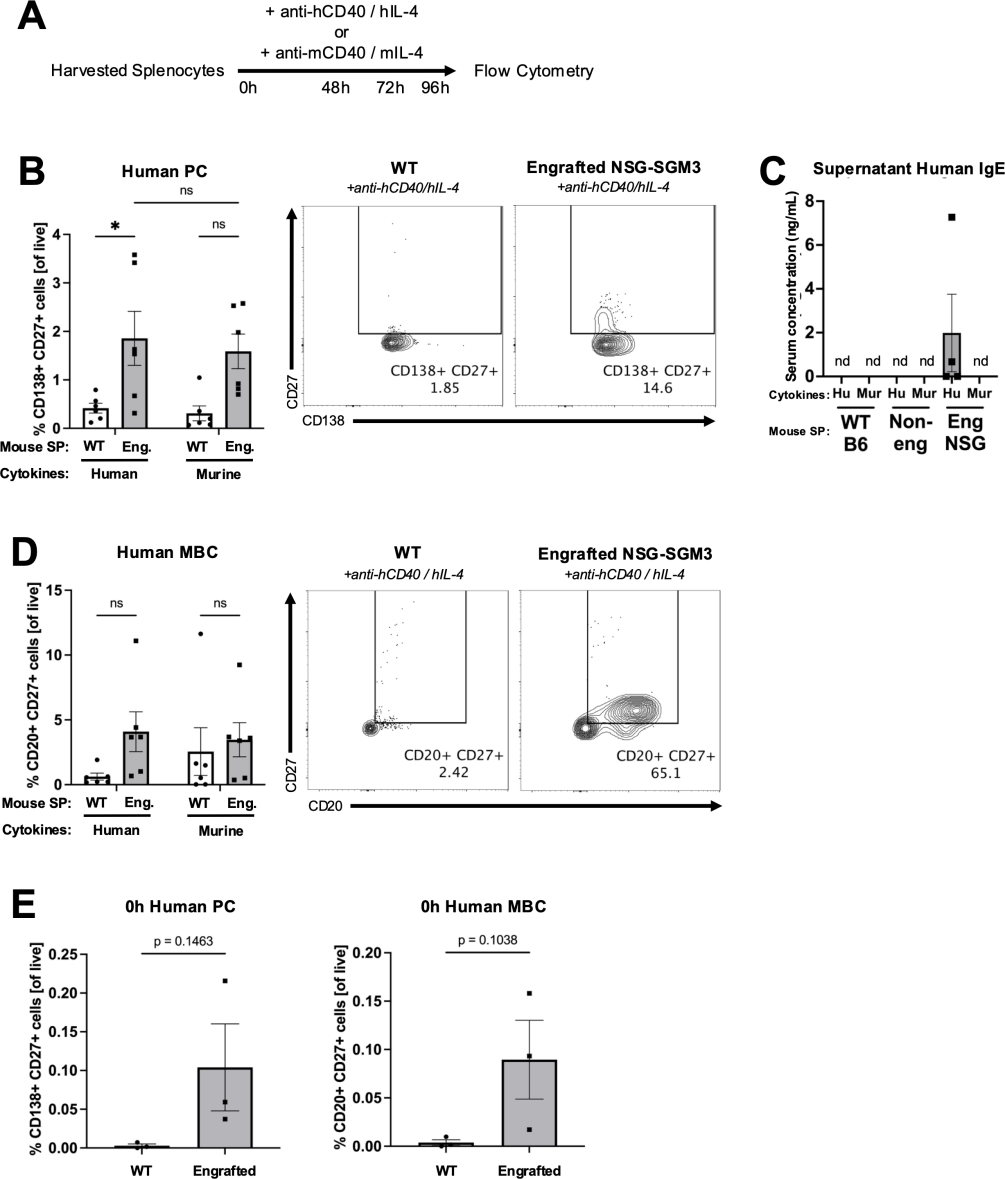
Human plasma cells and memory B cells expand from engrafted NSG-SGM3 splenocytes cultured with human Th2 cytokines *ex vivo.* **(A)** Experimental design for assessing *ex vivo* expansion of human and murine plasma cells (PC) or memory B cells (MBC) from bulk splenocytes (SP). Harvested splenocytes were cultured with anti-human CD40 and human IL-4 cytokines (anti-hCD40/hIL-4) or anti-murine CD40 and murine IL-4 cytokines (anti-mCD40/mIL-4) for 96 hours (h). **(B)** Percentage of and representative flow cytometry plots for CD138^+^ CD27^+^ human PC (of all live) from WT (circle) versus engrafted NSG-SGM3 ("Eng."; square/gray) splenocytes cultured for 96 hours with either human or murine cytokines. Values were calculated using cell counts of sequentially gated CD138^+^ CD19^var^, IgD^-^, CD23^-^, CD138^+^ CD27^+^ human PC, divided by all live cells (*n* = 6 mice/group, compiled from three independent experiments). **(C)** Concentration of human IgE in supernatants of *ex vivo* cultures of WT, non-engrafted NSG-SGM3, or engrafted NSG-SGM3 splenocytes cultured with either human or murine cytokines (*n* = 4 mice/group). **(D)** Percentage of and representative flow cytometry plots for CD20^+^ CD27^+^ human MBC (of all live) from WT versus engrafted NSG-SGM3 (square/gray) splenocytes cultured for 96 hours with either human or murine cytokines. Values were calculated using cell counts of sequentially gated CD19^+^ CD138^-^, IgD^-^, CD23^-^, CD20^+^ CD27^+^ human MBC, divided by all live cells (*n* = 6 mice/group, compiled from three independent experiments). **(E)** Baseline percentage of human PC and MBC for WT versus engrafted NSG-SGM3 (square/gray) splenocytes at zero hours prior to *ex vivo* culture. Values were calculated as discussed in **(B, D)** (*n* = 4 mice/group, compiled from two independent experiments). Error bars in **(B–E)** represent SEM. P values < 0.05 were considered significant (*p < 0.05; ns, not significant). Two-way ANOVA with post-hoc Šídák’s multiple comparisons test (full model mixed-effects analysis) was used for **(B, D)**, and unpaired Student’s *t-*test was used for **(E)**. See **Supplementary Figures S1-S3** and **Supplementary Table S2** for additional details on gating strategy and antibody staining for flow cytometry (SP, splenocytes; Hu, human cytokines, Mur, murine cytokines).

**Figure 3:**
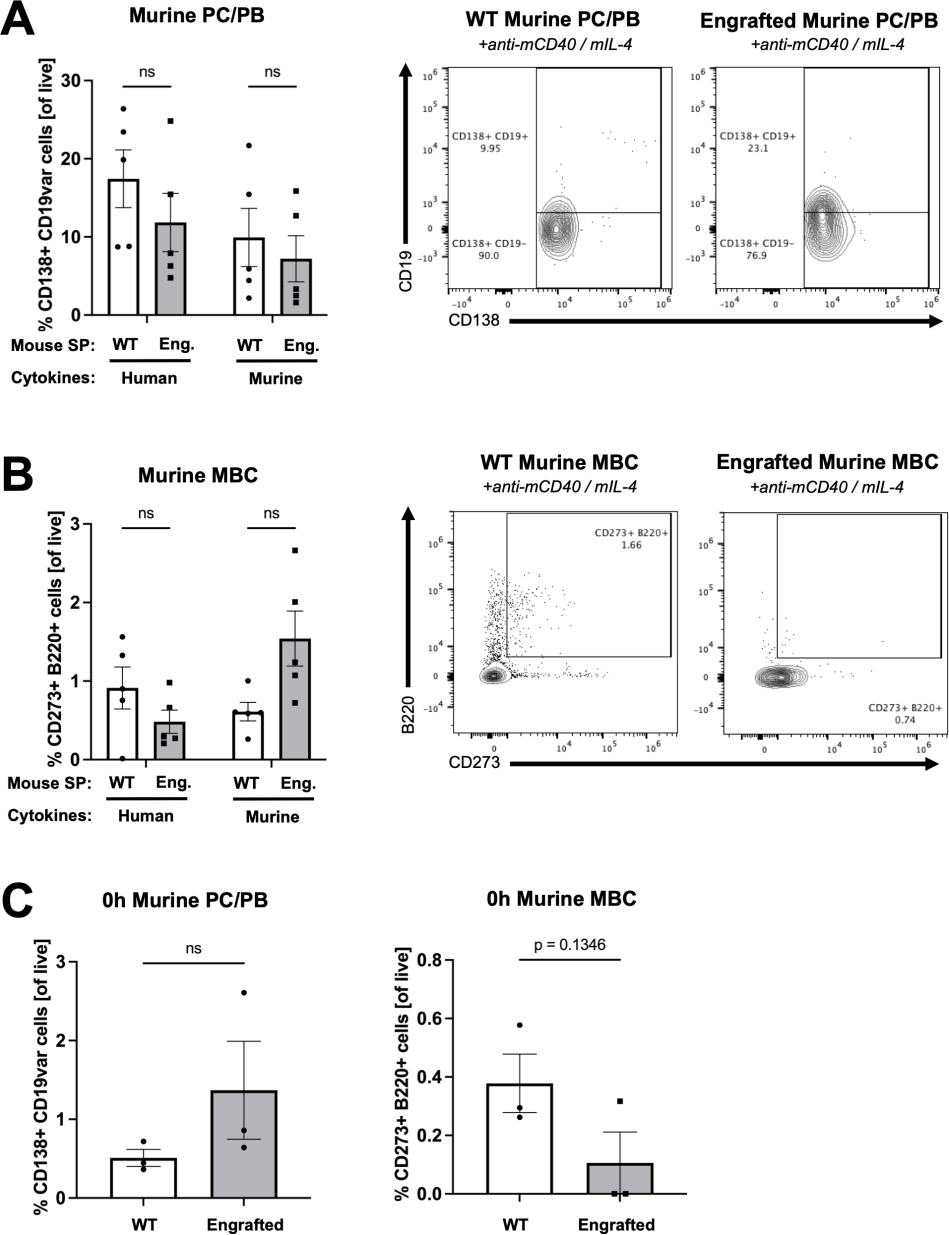
Human B cells demonstrate minimal expansion from engrafted NSG-SGM3 splenocytes cultured with murine cytokines *ex vivo*. **(A)** Percentage of and representative flow cytometry plots for CD138^+^ B220^lo^ murine PC and plasmablasts (PB) (of all live) from WT versus engrafted NSG-SGM3 ("Eng."; square/gray) splenocytes (SP) cultured for 96 hours with either human or murine cytokines. Values were calculated using cell counts of sequentially gated CD138^+^, IgD^-^ CD22^-^, GL-7^lo^, B220^lo^ murine PC (CD19^+/hi^) plus PB (CD19^lo/-^) cells, divided by all live cells (*n* = 6 mice/group, compiled from three independent experiments). **(B)** Percentage of and representative flow cytometry plots for CD273^+^ B220^+^ murine MBC (of all live) from WT versus engrafted NSG-SGM3 (square/gray) splenocytes cultured for 96 hours with human or murine cytokines. Values were calculated using cell counts of sequentially gated CD138^+^, IgD^-^ CD22^-^, GL-7^lo^, CD273^+^ B220^+^ murine MBC, divided by all live cells (*n* = 6 mice/group, compiled from three independent experiments). **(C)** Baseline percentage of murine PC/PB and MBC for WT versus engrafted NSG-SGM3 (square/gray) splenocytes at zero hours prior to *ex vivo* culture. Values were calculated as discussed in **(A, B)** (*n* = 4 mice/group, compiled from two independent experiments). Error bars represent SEM. P values < 0.05 were considered significant (ns, not significant). Two-way ANOVA with post-hoc Šídák’s multiple comparisons test (full model mixed-effects analysis) was used for **(A, B)**, and unpaired Student’s *t-*test was used for **(C)**. See **Supplementary Figures S1-S3** for additional details on gating strategy and antibody staining for flow cytometry (SP, splenocytes).

**Figure 4:**
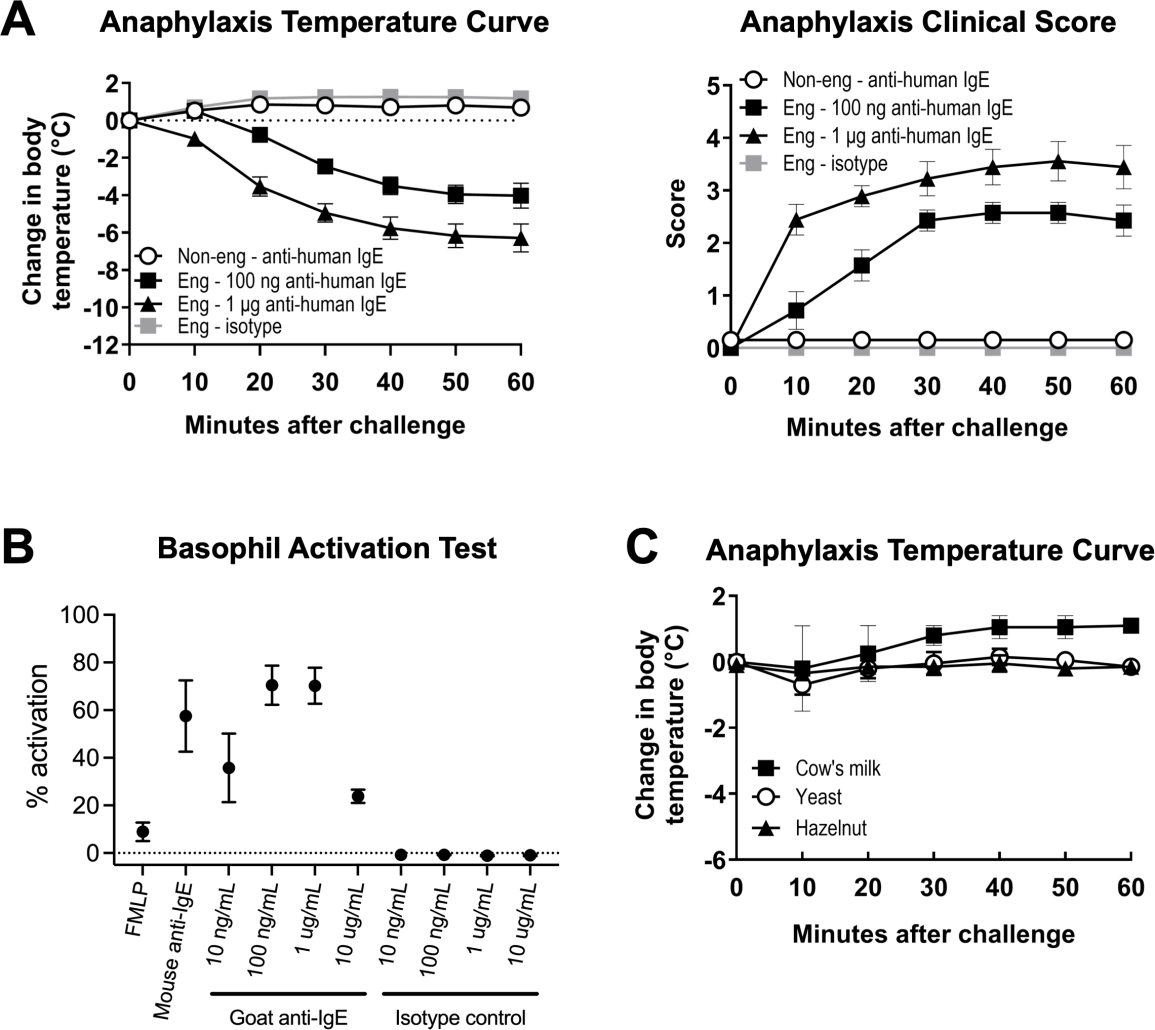
Engrafted NSG-SGM3 mice can undergo anaphylaxis with anti-human IgE alone but not by oral challenge with food allergen. **(A)** Change in body temperature (left panel) and clinical scores (right panel) as measures of passive systemic anaphylaxis (PSA) in engrafted NSG-SGM3 mice ("Eng") challenged with 100 ng anti-human IgE (black squares), 1 μg anti-human IgE (black triangles), or equivalent doses of isotype control (gray squares), versus non-engrafted NSG-SGM3 mice challenged with the same doses of anti-human IgE ("Non-eng"; white circles). Values represent mean ± SEM (*n* = 7–14 mice/group, compiled from five independent experiments). Clinical scores range from zero to five as follows: 0) asymptomatic; 1) scratching; 2) piloerection, facial edema; 3) labored breathing, reduced activity; 4) coma or unresponsiveness; and 5) death). **(B)** Percentage of basophil activation for whole blood basophils from healthy human donors activated for 30 minutes with 1 µM FMLP, 1 µg/mL mouse anti-IgE, or indicated concentrations of polyclonal goat anti-human IgE ("Goat anti-IgE") or goat polyclonal IgG isotype control ("Isotype control"). Values represent mean percentage of basophils with upregulated CD63 surface expression by flow cytometry (*n* = 3 human donors). **(C)** Change in body temperature as measure of systemic anaphylaxis for engrafted NSG-SGM3 mice challenged via oral gavage to foods to which they had positive specific IgE antibodies: cow’s milk (black squares), yeast (white circles), and hazelnut (black triangles). All clinical scores for food challenge experiments were 0. Values represent mean ± SEM (*n* = 2 mice for three foods each). Error bars represent SEM.

Each datapoint represents one sample from one mouse and error bars represent SEM in **Figures 1**–**3** unless otherwise stated. Data and error bars in **Figure 4** represent mean ± SEM unless otherwise stated. Statistical analyses were determined by unpaired Student’s *t-*test or two-way ANOVA with post-hoc Šídák’s multiple comparisons test (full model mixed-effects analysis), as specifically denoted in the figure captions and applied based on experimental design. P-values < 0.05 were considered statistically significant. Statistical tests and plotting were performed using GraphPad Prism 10 (GraphPad Software, Inc).

## 3 Results

### 3.1 Naïve engrafted NSG-SGM3 mice produce abundant circulating human antibodies, including IgE, in the absence of active sensitization

The sera of NSG-SGM3 mice were first analyzed for human antibody titers, demonstrating that naïve engrafted NSG-SGM3 mice without prior marrow ablation produced easily detectable quantities of human IgM, IgG, IgA, and IgE antibodies without active sensitization to antigens, as compared to no detectable amounts from non-engrafted NSG-SGM3 or wildtype C57BL/6J (WT B6) controls (**Figure 1A**). Similar concentrations of serum total IgE were seen within batches of NSG-SGM3 mice engrafted with the same donor HSCs, and IgE concentrations also correlated better by donor HSC than by engraftment efficiency determined by circulating human CD45^+^ count. Of note, even the lowest IgE levels were remarkably several-fold more abundant than that of the average human patient (typically below 200–240 ng/mL for healthy non-atopic humans) (25, 26), while the levels of human IgM, IgG, and IgA were sub-physiologic by several folds (27–29). These human antibody concentrations highlight basal human antibody production in our naïve and otherwise unsensitized humanized mice raised in specific-pathogen free facilities.

We next investigated the landscape of circulating human cytokines, particularly those necessary for human B cell antibody class switching. NSG-SGM3 mice exhibited comparable serum levels of human GM-CSF and IL-3 regardless of the presence or absence of HSC engraftment or the efficiency of HSC engraftment determined by circulating human CD45^+^ count (**Figure 1B**), reflecting constitutive production as previously described (10, 30). NSG-SGM3 mice also produced substantial human SCF (**Figure 1B**), with engrafted mice expressing similar levels to those previously reported for irradiated NSG-SGM3 BLT mice engrafted with fetal liver-derived HSCs (10). However, only engrafted NSG-SGM3 mice exhibited easily detectable serum levels of human IL-4 and IL-13 (**Figure 1C**), which are cytokines essential for type II immunity and IgE class switch recombination, as well as physiologic ranges of human Th1 cytokines IL-6, interferon gamma (IFN-γ), and tumor necrosis factor alpha (TNF-α) (**Figure 1D**) (31–34). Surprisingly, both engrafted and non-engrafted NSG-SGM3 mice produced large amounts of murine IL-4 from residual murine immune cells (**Figure 1C**). In contrast, WT B6 mice used as controls did not have detectable serum murine IL-4, consistent with prior reports for this mouse strain which is known to produce less IL-4 than other strains due to fewer invariant natural killer T cells (33, 35). These data thus confirm that engrafted human HSCs in NSG-SGM3 mice without prior ablation can differentiate into functional human immune cells that produce human Th2 cytokines to support B cell class switching and IgE production.

### 3.2 Human IgE from engrafted NSG-SGM3 mice is polyclonal and recognizes a diverse array of allergens

To further profile the functional roles of human IgE produced by humanized NSG-SGM3 mice, we sought to determine the range of allergen specificity of their serum human IgE. We utilized the ALEX^2^ multiplex chip to test human IgE reactivity to 295 clinically relevant molecular and extract-based allergens, including aeroallergens (e.g. grasses, trees, animal dander), common plant-derived and animal-derived food allergens, and stinging insect venoms. Pooled serum samples from engrafted NSG-SGM3 mice, which again demonstrated considerable total human IgE levels (**Figure 1E**), revealed a diverse profile of allergen specificities (**Table 1**; **Supplementary Table S3**). Low levels of specific IgE antibodies, all below 2 kUA/L, were detected across seven of eight total broad categories of allergens (plant-derived food; animal-derived food; pollen; mite; insect/venom; animal origin; microorganism/spore; other). The highest specific IgE levels observed were either against foods not present in the mouse chow diet (**Supplementary Table S1**) or against allergens otherwise highly unlikely to be encountered in the specific-pathogen free mouse facility environment, including millet *Pan m* (0.17–1.35 kUA/L), egg yolk *Gal d_yolk_* (0.13–0.22 kUA/L), cashew *Ana o* (0.22–0.26 kUA/L), and cottonwood (0.71–0.83 kUA/L) (**Table 1**; **Supplementary Table S3**). This diverse and broad range of allergen specificities therefore suggests that even without deliberate active sensitization, NSG-SGM3 mice produce low basal levels of functional polyclonal human IgE specific to diverse allergens, likely including numerous others not tested for by the ALEX^2^ chip, as opposed to one monoclonal IgE antibody specific to a singular allergen.

**Table 1:** Engrafted NSG-SGM3 mice produce polyclonal human IgE specific to a diverse array of allergens.

Plant food							Animal food						
Type	Subtype	Allergen	E/M	P_1	P_2	P_3	Type	Subtype	Allergen	E/M	P_1	P_2	P_3
Legume	Peanut	Ara h 1	M	≤0.10	≤0.10	0.12	Milk	Camel, milk	Cam d	E	≤0.10	0.36	0.34
		Ara h 8	M	≤0.10	0.12	0.16	Egg	Egg white	Gal d_white	E	0.12	0.12	0.14
	Chickpea	Cic a	E	≤0.10	0.12	0.18		Egg yolk	Gal d_yolk	E	0.13	0.19	0.22
	Soy	Gly m 4	E	≤0.10	0.11	≤0.10	Meat	House cricket	Ach d	E	≤0.10	0.14	0.13
	Lentil	Len c	E	≤0.10	≤0.10	0.14		Rabbit, meat	Ory_ meat	E	≤0.10	≤0.10	0.16
	Cashew	Ana o	E	≤0.10	0.26	0.22	Seafood	Herring worm	Ani s 1	M	0.18	0.16	≤0.10
	Pecan	Car i	E	≤0.10	0.18	0.24	**Pollen**						
	Walnut	Jug r 1	M	≤0.10	0.17	0.23	**Type**	**Subtype**	**Allergen**	**E/M**	**P_1**	**P_2**	**P_3**
		Jug r 6	M	≤0.10	≤0.10	0.17	Grass pollen	Timothy grass	Phl p 1	M	0.23	0.24	0.20
	Maca-damia	Mac inte	E	≤0.10	0.10	0.16		Common reed	Phr c	E	≤0.10	≤0.10	0.16
Seed	Pumpkin seed	Cuc p	E	≤0.10	0.34	0.86	Tree pollen	Tree of heaven	Ail a	E	≤0.10	0.17	0.29
Spice	Paprika	Cap a	E	≤0.10	0.34	0.48		Cypress	Cup a 1	M	≤0.10	≤0.10	0.12
	Parsley	Pet c	E	≤0.10	≤0.10	0.15			Cup s	E	≤0.10	0.25	0.34
Cereal	Oat	Ave s	E	≤0.10	0.17	0.15		Beech	Fag s 1	M	≤0.10	0.10	0.13
	Quinoa	Che q	E	≤0.10	≤0.10	0.16		London plane tree	Pla a 1	M	≤0.10	0.10	0.14
	Common buck-wheat	Fag e	E	≤0.10	0.20	0.25		Cotton-wood	Pop n	E	≤0.10	0.71	0.83
	Barley	Hor v	E	≤0.10	0.21	0.16		Elm	Ulm c	E	≤0.10	0.23	0.34
	Lupine seed	Lup a	E	≤0.10	0.15	0.21	Weed pollen	Russian thistle	Sal k	E	≤0.10	0.17	0.16
	Rice	Ory s	E	0.21	0.28	0.16	**Insect/venom**						
	Millet	Pan m	E	0.17	1.04	1.35	**Type**	**Subtype**	**Allergen**	**E/M**	**P_1**	**P_2**	**P_3**
	Cultivated rye	Sec c_flour	E	≤0.10	0.15	0.22	Wasp venom	Paper wasp venom	Pol d	E	≤0.10	0.22	0.18
	Wheat	Tri a 19	M	0.11	0.13	0.21		Wasp venom	Ves v	E	≤0.10	0.13	≤0.10
Fruit	Kiwi	Act d 1	M	≤0.10	0.28	0.40	**Mite**						
		Act d 2	M	≤0.10	0.22	0.19	**Type**	**Subtype**	**Allergen**	**E/M**	**P_1**	**P_2**	**P_3**
Vegetable	Onion	All c	E	≤0.10	0.18	0.21	House dust mite	European house dust mite	Der p 7	M	≤0.10	0.15	≤0.10
	Potato	Sol t	E	≤0.10	0.12	0.19			Der p 21	M	≤0.10	0.11	≤0.10
**Microorganism/Spore**							Storage mite	Blomia tropicalis	Blo t 5	M	≤0.10	0.17	≤0.10
**Type**	**Subtype**	**Allergen**	**E/M**	**P_1**	**P_2**	**P_3**			Blo t 10	M	0.10	≤0.10	≤0.10
Yeast	Yeast	Sac c	E	0.15	0.10	0.11		Glycy-phagus domesticus	Gly d 2	M	≤0.10	0.18	0.14
**Other**							**Legend**						
**Type**	**Subtype**	**Allergen**	**E/M**	**P_1**	**P_2**	**P_3**	Negative/Uncertain		≤0.10 kUA/L		Moderate IgE		1-5 kUA/L
Latex	Latex	Hev b 3	M	≤0.10	≤0.10	0.10	Very low IgE		0.10-0.30 kUA/L		High IgE		5-15 kUA/L
Ficus	Weeping fig	Fic b	E	0.10	0.23	≤0.10	Low IgE		0.30-1 kUA/L		Very high IgE		> 15 kUA/L

List of positive (>0.10 kUA/L) molecular and extract-based allergen specificities of engrafted NSG-SGM3-produced human IgE. Individual allergens are clustered hierarchically under broad allergen categories and types. Numbers indicate serum human IgE (kUA/L) for three pooled samples of five engrafted NSG-SGM3 mice each (P_1, P_2, P_3), as determined by ALEX^2^ ELISA-based IgE multiplex assay. Cutoffs for IgE concentration and severity levels/categories follow the default cutoffs listed for the standard ALEX^2^ chip analysis, with positive signal for very low IgE level >0.10 kUA/L as such: white, negative/uncertain IgE level (≤0.10 kUA/L); light yellow, very low IgE (0.10-0.30 kUA/L); green-yellow, low IgE (0.30-1 kUA/L); green, moderate IgE (1-5 kUA/L); dark green, high IgE (5-15 kUA/L); black, very high IgE (>15 kUA/L) (E, allergen extract; M, recombinant molecular allergen).

### 3.3 Human CD40L and IL-4 expand human plasma cells and memory B cells in engrafted NSG-SGM3 splenocytes *ex vivo*

Next, we aimed to validate whether NSG-SGM3 mice can support the differentiation and maturation of engrafted human CD34^+^ HSCs into human IgE-producing B cells. We first stimulated splenocytes from engrafted NSG-SGM3, non-engrafted NSG-SGM3, or WT B6 mice *ex vivo* using either human or murine stimulatory CD40L and IL-4 cytokines (**Figure 2A**) (22, 23). We then analyzed cells by flow cytometry for the expansion of human versus murine plasma cells (PC) and memory B cells (MBC) (**Supplementary Figures S1-S3**) (18, 36–39).

As compared to WT splenocytes, engrafted NSG-SGM3 splenocytes stimulated with human CD40L and IL-4 cytokines *ex vivo* for 96 hours exhibited a significantly increased percentage of human CD138^+^ CD27^+^ cells, indicating relative enrichment of antibody-producing human PC (**Figure 2B**). While there was mild but non-significant expansion of human PC in response to murine cytokines (**Figure 2B**), suggestive of low/minimal interspecies cross-reactivity, human IgE antibodies were detected in the culture supernatants of only engrafted NSG-SGM3 splenocytes stimulated with human (but not murine) cytokines (**Figure 2C**). In contrast, the percentage of murine PC and younger plasmablasts (PB) in response to human or mouse stimulatory cytokines was similar between engrafted NSG-SGM3 mice and WT mice (**Figure 3A**). Notably, non-engrafted NSG-SGM3 splenocytes were poor controls for flow cytometry due to significant autofluorescence at baseline and high cell death during splenocyte harvest and throughout *ex vivo* culture, which contributed to significant non-specific and falsely positive fluorescent signal (**Supplementary Figure S4**). Even so, the undetectable levels of human IgE from culture supernatants of non-engrafted NSG-SGM3 mice stimulated with human or murine cytokines suggest that human IgE-secreting PC were not expanded (**Figure 2C**), as expected given their lack of engrafted human HSCs. Therefore, engrafted NSG-SGM3 mice harbor a Th2 cytokine-responsive population of human PC that can secrete human IgE.

Consistent with our PC findings, engrafted NSG-SGM3 stimulated with human cytokines also exhibited an increase in the percentage of human CD20^+^ CD27^+^ cells as compared to WT splenocytes, which reflects expansion of human MBC (**Figure 2D**), despite comparable percentages of murine MBC (**Figure 3B**). In addition, at baseline prior to *ex vivo* culture, human PC and MBC were detected in engrafted NSG-SGM3 mice, albeit at rare and low percentages, but were undetectable in WT splenocytes (**Figure 2E**). In contrast and as expected, at baseline prior to culture, engrafted NSG-SGM3 mice had a comparable percentage of murine PC/PB and mildly decreased percentage of murine MBC as compared to WT mice (**Figure 3C**). These data are suggestive of the presence of rare and possibly resting or lowly-active maintenance populations of human PC and MBC in engrafted NSG-SGM3 mice, which can expand upon Th2 cytokine stimulation even with present residual murine immune cells. Our *ex vivo* findings also support our data on abundant human antibody production in naïve engrafted NSG-SGM3 mice (**Figure 1A**), by highlighting putative upstream human HSC-derived cells which can mature into and expand human PC and MBC populations in response to Th2 cytokines to help sustain the production of basal human IgE.

### 3.4 Engrafted NSG-SGM3 mice can undergo anaphylaxis following administration of anti-human IgE antibody but not by challenge with food allergen

Lastly, we explored the functionality of human IgE produced by engrafted NSG-SGM3 mice. To understand the anaphylaxis-inducing potential of the polyclonal human IgE and confirm binding of this human IgE to surface FcϵRI receptors on human mast cells and basophils, we utilized a one-step model involving systemic challenge with a single dose of goat anti-human IgE antibody. Remarkably, a single intraperitoneal injection of this goat IgG specific to human IgE into engrafted NSG-SGM3 mice induced a significant dose-dependent decrease in core body temperature and increase in anaphylaxis clinical scores (**Figure 4A**). In contrast, non-engrafted NSG-SGM3 mice given anti-human IgE and engrafted NSG-SGM3 mice given goat IgG isotype control did not exhibit clinical reactivity (**Figure 4A**). We further confirmed the lack of interspecies binding of goat IgG to murine or human Fcγ receptors using *in vitro* basophil activation assays (**Figure 4B**). As murine FcϵRI receptors cannot bind human IgE (40), our data demonstrate that the polyclonal human IgE produced by humanized NSG-SGM3 mice is physiologically functional and can induce human cell-dependent type I hypersensitivity responses such as anaphylaxis by direct targeting.

We further investigated whether engrafted NSG-SGM3 mice with low levels of food allergen-specific IgE antibodies could undergo anaphylaxis in response to specific food allergen challenge. Engrafted NSG-SGM3 mice did not exhibit clinical reactivity after oral gavage with cow’s milk (serum anti-Bos d 4-IgE of 0.13 and 0.31 kUA/L, respectively), yeast (anti-Sac c-IgE of 0.43 and 0.65), or hazelnut (anti-Cor a 11-IgE of 0.19 and 0.41) (**Figure 4C**). Together, these data suggest that human IgE in humanized NSG-SGM3 mice do harbor functional Fc receptors but, at least with such low levels of food-specific IgE, are not able to elicit clinical anaphylaxis by oral challenge with food allergen.

## 4 Discussion

We herein demonstrate that humanized NSG-SGM3 mice engrafted with human HSCs spontaneously produce all human antibody isotypes (**Figure 1A**), even without prior marrow ablation or human lymphoid tissue implantation. To our knowledge, this is the first report that NSG-SGM3 mice can produce human IgE (**Figures 1A, E**). This is consistent with prior reports of human IgM and IgG production in BLT mice and NSG-SGM3 mice engrafted after irradiation (4, 18). Furthermore, we demonstrate that the human IgE is polyclonal and recognizes a diverse set of allergens (**Table 1**; **Supplementary Table S3**). This finding further highlights how human HSC engraftment alone is actually “self-sufficient” to generate human Th2 cytokines needed for human IgE production despite a still-present residual murine background (**Figure 1C**). Thus, our novel findings characterize the spontaneous production of a polyclonal IgE repertoire in an otherwise unsensitized NSG-SGM3 humanized mouse.

Additionally, we identify putative human CD138^+^ CD27^+^ plasma cell and CD20^+^ CD27^+^ memory B cell populations expanded by Th2 cytokines to facilitate IgE secretion (**Figures 2B–D**), offering a novel humanized mouse model for the study of human IgE B cell biology. As human PC and MBC tend to reside in lymphoid structures (41), it is perhaps surprising that an unablated NSG-SGM3 mouse with only murine lymphoid structures can adequately support human B cell survival and differentiation from engrafted human HSCs, let alone functional human IgE production. In addition to human Th2 cytokines, other cross-reactive signals impacting human PC and MBC expansion are likely also released *in vivo* by host murine lymphoid and stromal tissues, even if differences in mouse and human lymphoid organ structure or cellular composition are driving different signaling molecule repertoires. Of note, our culture model using bulk splenocytes rather than purified B cells may better capture the full spectrum of cross-reactive murine signals such as pro-apoptotic factors (**Figure 2A**), given that human PC and MBC were indeed detected from engrafted NSG-SGM3 mice (**Figures 2B–D**). Additional homing sites in other murine lymphoid tissues may also exist. It would be interesting to characterize the trafficking patterns of human PC and MBC and any differing murine-derived signaling factors in our NSG-SGM3 model as compared to traditional BLT mice. This is likely difficult to study, however, especially as IgE^+^ B cells are exceedingly rare and thus difficult to track without IgE-inducing triggers such as allergen sensitization, glucocorticoid exposure, or germ-free diet from a young age (23, 39, 42).

It is also of interest to consider the source and physiologic implications of IgE memory in our model. As our humanized NSG-SGM3 mice produce basal polyclonal IgE found in blood and presumably also bound to FcϵRI receptors on mast cells and basophils (**Figure 1A** and **4A**), there is likely constant low-level repopulation of multiple clones of human IgE-secreting cells to maintain basal secretion of polyclonal IgE. Data on the source of IgE memory in humans favor a population of IgG MBC which can re-differentiate into IgG plasma cells and subsequently class switch to and secrete IgE (39, 41, 43–50), though human IgE^+^ MBC that directly re-differentiate into IgE-secreting PC may also exist (51, 52). The former hypothesis is bolstered by data showing that chronic IgE B cell receptor signaling is self-constraining, which not only regulates IgE production by intrinsically restricting the lifespans of IgE^+^ B cells and PC, but likely also prohibits differentiation into IgE^+^ MBC (46–48, 50). While we demonstrated human IgE secretion by engrafted NSG-SGM3 splenocytes stimulated *ex vivo* with Th2 cytokines (**Figure 2C**), the signaling kinetics of and the downstream impact on human B cell maturation *in vivo* for IgE^+^ B cell receptors specifically still need to be explored.

Additionally, given that older NSG-SGM3 mice can develop HLH/MAS-like pancytopenia (14–17), a component of inadequate or even absent immune regulation may be involved in priming these mice for IgE production. Although irradiated and engrafted NSG-SGM3 mice have increased functional regulatory T cells (10), gut dysbiosis in the setting of a murine-immunodeficient host or other factors known to drive high IgE levels in mice could further contribute to these human IgE levels above typical non-atopic levels (23, 42, 53, 54). Further investigations into cell-cell circuits and other physiologic and/or immune regulatory states affecting human IgE production and functionality in NSG-SGM3 mice and other humanized mouse models are warranted.

One limitation of our work includes the fact that we have not identified that human IL-4 is necessary for production of human IgE, for example by administering an anti-IL-4 blocking antibody. Furthermore, the exact cellular sources of human IL-4 presumably driving human IgE class switching in our model remain unclear. The most likely candidates are human basophils, which are greatly expanded in response to high levels of IL-3, SCF, and GM-CSF from the human transgenes (8). Other possible but likely minor contributors include human T follicular helper cells (Tfh) and type II innate lymphoid cells (ILC2). On the other hand, murine IL-4, which is secreted by residual murine granulocytes in NSG-SGM3 mice (8), is thought to be species-specific and not cross-reactive for human B cells (55, 56). In line with this, our *ex vivo* data clearly demonstrate that murine Th2 cytokines do not significantly expand human CD138^+^ CD27^+^ PC and CD20^+^ CD27^+^ MBC (**Figures 2B, D**) or drive IgE production (**Figure 2C**) in engrafted NSG-SGM3 splenocytes. Though low cross-reactivity from murine IL-4 cannot be fully ruled out, the effects are likely minimal and human IL-4 remains as the most likely driving signal of human IgE production in humanized NSG-SGM3 mice.

We also demonstrate that the NSG-SGM3 basal IgE repertoire recognizes an array of human-relevant allergens despite the lack of intentional active allergen sensitization (**Table 1**; **Supplementary Table S3**). These low specific IgE titers likely reflect low-level sensitization without allergic reactivity, given that the mice do not appear to react to food allergen epitopes. Although anaphylaxis was induced by cross-linking IgE directly via administration of anti-human IgE antibody (**Figure 4A**), no clinical reactivity was observed during oral challenge with multiple food allergens to which the mice had never before been exposed (**Figure 4C**). This lack of clinical reactivity could be due to the very low specific IgE titers, often in the range of 0.1-0.3 kUA/L, which are below the typical clinical cut-off of 0.3 kUA/L for diagnostics in humans. For a small subset of food allergens, such as wheat and soy, it is possible that the presence of these foods in the daily diet of these mice (**Supplementary Table S1**) may have induced a desensitized state. Of note, however, we only tested three easily accessible food allergens for oral allergen challenge, and additional reactive allergens of clinical significance may not have been captured within the 295 tested allergens (**Supplementary Table S3**). Still, these low titers could reflect the observation that many human patients also produce basal polyclonal serum IgE to various allergens without symptomology upon exposure (57–59).

However, the full mechanism behind the production and function of this polyclonal IgE is still unclear. Of note, our humanized mice were introduced to mouse chow in young adulthood prior to engraftment and also received multipotent human HSCs rather than differentiated B cells. As the positive allergen-specific IgE titers varied between samples, which were pooled from mice receiving different donor HSCs, it is possible that features specific to each donor patient could dictate the particular repertoire of allergen specificities. For example, epigenetic programming of donor patient HSCs prior to extraction (60–63), perhaps in response to real-world or *in utero* allergen exposures or other immunomodulatory signals (e.g. from gut microbiota) sustained earlier by the donor patient (42, 62, 64), may potentially skew the clonality of the IgE repertoire, though this is not well-studied. Other mechanisms modulating IgE function may also be involved. For example, the anaphylactic capacity of IgE can be altered by glycosylation modifications (65–67). Widespread expression of the low-affinity IgE receptor CD23 on abundant immune cells can also act like an IgE “sink” to mildly alter IgE availability for binding to FcϵRI on granulocytes and thus downstream granulocyte function (68–70).

Despite polyclonal reactivity of the human IgE to multiple allergens (**Table 1**), different IgE antibodies within the NSG-SGM3 IgE repertoire could have variable affinity to their allergen such that some IgE antibodies are individually inadequate to induce anaphylaxis by allergen challenge. Typically, high-affinity anaphylactic IgE is thought to require sequential switching of IgG B cells to IgE B cells in germinal centers for somatic hypermutation and affinity maturation (71, 72). In contrast, lower-affinity and broadly-specific “natural” IgE, produced by direct switching from IgM to IgE B cells without significant affinity maturation (23, 73, 74), is thought to play homeostatic immune roles such as protection from anaphylaxis, cancer surveillance, and skin barrier defenses (23, 75, 76). It is also possible that the polyclonal human IgE repertoire of NSG-SGM3 mice could functionally act like broadly- or non-specific IgE in bulk. This would be consistent with the notion that natural IgE functions in immune protection and surveillance, which may modulate or even compensate in part for the known immune-dysregulated background of NSG-SGM3 mice.

In all, our work uncovers the utility of humanized NSG-SGM3 mice without prior ablation as a humanized mouse model for studying human antibody and B cell biology and for elucidating the physiologic roles of a basal polyclonal antibody repertoire. In particular, this is the first report that naïve NSG-SGM3 mice spontaneously generate polyclonal and functional IgE, which may reflect in part why many human subjects have basal levels of allergen-specific IgE without significant clinical reactivity. Our data also reveal additional novel insights into the dynamics of human B cell maturation, differentiation, and proliferation, in particular for Th2 cytokine-responsive lymphocytes that can facilitate the maintenance of a basal and polyclonal IgE repertoire even without prior sensitization.

## Data Availability

The raw data supporting the conclusions of this article will be made available by the authors, without undue reservation.
