# Supplementary material for: Engrafted NSG-SGM3 humanized mice spontaneously produce human immunoglobulins including IgE
Source: Front Immunol. 2025 Aug 25;16:1628194. doi: 10.3389/fimmu.2025.1628194 (PMC12414937; doi:10.3389/fimmu.2025.1628194)

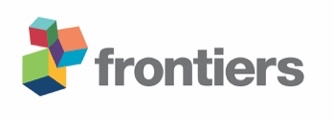


Supplementary Material

1. **Supplementary Table S1**

**Supplementary Table S1: Components of mouse chow.** Table of primary ingredients in descending order of inclusion (left) and calculated nutrient profile (middle and right) for Tekland Global 18% protein extruded rodent diet (Inotiv Inc.).

| **Tekland Global 18% Protein Extruded Rodent Diet** **(Inotiv Inc.)** | | | | |
| --- | --- | --- | --- | --- |
| **Primary Ingredients** **(in Descending Order)** | **Macronutrients** | | **Amino Acids** | |
| 1. Ground wheat | Crude protein | 18.4% | Aspartic acid | 1.4% |
| 2. Ground corn | Fat (ethar extract) | 6.0% | Glutamic acid | 3.4% |
| 3. Wheat middlings | Fat (acid hydrolysis) | 7.0% | Alanine | 1.1% |
| 4. Dehulled soybean meal | Carbohydrate (available) | 44.2% | Glycine | 0.8% |
| 5. Corn gluten meal | Crude fiber | 3.8% | Threonine | 0.7% |
| 6. Soybean oil | Neutral detergent fiber | 14.7% | Proline | 1.6% |
| 7. Calcium carbonate | Ash | 5.5% | Serine | 1.1% |
| 8. Dicalcium phosphate | Energy density | 3.1 kcal/g | Leucine | 1.8% |
| 9. Brewers dried yeast | Calories from protein | 24% | Isoleucine | 0.8% |
| 10. Iodized salt | Calories from fat | 18% | Valine | 0.9% |
| 11. L-lysine | Calories from carbs. | 58% | Phenylalanine | 1.0% |
| 12. DL-methionine | **Minerals** | | Tyrosine | 0.6% |
| 13. Calcium propionate | Calcium | 1.0% | Methionine | 0.6% |
| 14. Choline chloride | Phosphorus | 0.7% | Cystine | 0.3% |
| 15. Menadione sodium bisulfite complex (vitamin K activity) | Non-phytate phosphorus | 0.4% | Lysine | 1.1% |
| 16. Magnesium oxide | Sodium | 0.2% | Histidine | 0.4% |
| 17. Vitamin E acetate | Potassium | 0.6% | Arginine | 1.0% |
| 18. Calcium pantothenate | Chloride | 0.4% | Tryptophan | 0.2% |
| 19. Thiamin mononitrate | Magnesium | 0.2% | **Vitamins** | |
| 20. Manganous oxide | Zinc | 70 mg/kg | Vitamin A | 30.0 IU/g |
| 21. Niacin | Manganese | 100 mg/kg | Vitamin D_3_ | 2.0 IU/g |
| 22. Ferrous sulfate | Copper | 15 mg/kg | Vitamin E | 135 IU/kg |
| 23. Zinc sulfate | Iodine | 6 mg/kg | Vitamin K_3_ (menadione) | 100 mg/kg |
| 24. Zinc oxide | Iron | 200 mg/kg | Vitamin B_1_ (thiamin) | 117 mg/kg |
| 25. Riboflavin | Selenium | 0.23 mg/kg | Vitamin B_2_ (riboflavin) | 27 mg/kg |
| 26. Vitamin A acetate | **Fatty Acids** | | Vitamin B_3_ (niacin / nicotinic acid) | 115 mg/kg |
| 27. Pyridoxine hydrochloride | C16:0 Palmitic | 0.7% | Vitamin B_6_ (pyridoxine) | 26 mg/kg |
| 28. Copper sulfate | C18:0 Stearic | 0.2% | Vitamin B_12_ (cyanocobalamin) | 0.15 mg/kg |
| 29. Vitamin B_12_ supplement | C18:1ω9 Oleic | 1.2% | Biotin | 0.90 mg/kg |
| 30. Folic acid | C18:2ω6 Linoleic | 3.1% | Folate | 9 mg/kg |
| 31. Calcium iodate | C18:3ω3 Linolenic | 0.3% | Choline | 1200 mg/kg |
| 32. Biotin | Total saturated | 0.9% | **Other** | |
| 33. Vitamin D_3_ supplement | Total monounsaturated | 1.3% | Cholesterol | N/A |
| 34. Cobalt carbonate | Total polyunsaturated | 3.4% |  |  |

1. **Supplementary Table S2**

**Supplementary Table S2: List of flow cytometry antibodies and reagents for *ex vivo* splenocyte cultures.** Antibodies for detecting human cells (anti-human panel; plasma cells [PC] versus memory B cells [MBC]), antibodies for detecting murine cells (anti-mouse panel; PC plus plasmablasts [PB] versus MBC), and miscellaneous flow cytometry reagents are listed with corresponding clones, alternative names if appropriate, dilution factors, catalog number, and company.

| **Anti-Human Panel** | | **Anti-Mouse Panel** | |
| --- | --- | --- | --- |
| CD45R/B220 APC/Cyanine7  Rat anti-mouse/human (clone RA3-6B2)  1:200 dilution | 103223  BioLegend | CD45R/B220 APC/Cyanine  anti-mouse/human (clone RA3-6B2)  1:200 dilution | 103223  BioLegend |
| CD19 Alexa Fluor 700  Mouse anti-human (clone SJ25C1)  1:200 dilution | 363033  BioLegend | CD19 Alexa Fluor 700  Rat anti-mouse (clone 6D5)  1:200 dilution | 115527  BioLegend |
| CD138 PE  Mouse anti-human (clone MI15)  1:200 dilution | 561704  BD Pharmingen | CD138 PE  Rat anti-mouse (clone 281-2)  1:200 dilution | 553714  BD Pharmingen |
| CD20 PerCP-Cy5.5  Mouse anti-human (clone 2H7)  1:200 dilution | 560736  BD Pharmingen | GL7 antigen PerCP/Cyanine5.5  Rat anti-mouse/human (clone GL7)  1:200 dilution | 144609  BioLegend |
| IgD FITC  Mouse anti-human (clone IA6-2)  1:200 dilution | 562023  BD Pharmingen | IgD FITC  Rat anti-mouse (clone 11-26c.2a)  1:200 dilution | 553439  BD Pharmingen |
| APC CD23  Mouse anti-human (clone EBVCS-5)  1:200 dilution | 338513  BioLegend | CD22 APC  Rat anti-mouse (clone OX-97)  1:200 dilution | 126109  BioLegend |
| CD27 BV421  Mouse anti-human (clone M-T271)  1:200 dilution | 562514  BD Horizon | CD273 BV421  Rat anti-mouse (clone TY25)  1:200 dilution | 107219  BioLegend |
|  |  |  |  |
|  |  |  |  |
| **Other Antibodies/Stains** | | **Compensation Beads** | |
| LIVE/Dead Fixable Aqua  1:800 dilution | L34966  Invitrogen | UltraComp ebeads Compensation Beads  Diluted per manufacturer protocol | 01-222-42  Invitrogen |
| Purified rat anti-mouse CD16/CD32  (mouse BD Fc block)  1:10 dilution | 553142  BD Pharmingen | ArC Amine Reactive Beads  Diluted per manufacturer protocol | A10346A  Invitrogen |
| Human BD Fc block  1:10 dilution | 564219  BD Pharmingen | ArC Amine Negative Beads  Diluted per manufacturer protocol | A10346b  Invitrogen |
| True-Stain Monocyte Blocker  1:10 dilution | 426102  BioLegend |  |  |

1. **Supplementary Table S3**

**Supplementary Table S3: Full list of molecular and extract-based allergen specificities of engrafted NSG-SGM3-produced human IgE.** Pooled sera from engrafted mice were analyzed by the ALEX^2^ multiplex chip, which contains 295 allergens that are clinically relevant to human allergic disease (aeroallergens, foods, insect venoms), including all 11 established cross-reactive allergen families: profilins; PR10-like; non-specific lipid transfer proteins; serum albumins; tropomyosins; polcalcins; lipocalins; seed storage proteins; oleosins; parvalbumins. Individual allergens in the table are clustered hierarchically under broad allergen categories and types. Numbers indicate serum human IgE (kUA/L) for three pooled samples of five engrafted NSG-SGM3 mice each (P_1, P_2, P_3). Cutoffs for IgE concentration and severity levels/categories follow the default cutoffs listed for the standard ALEX^2^ chip analysis, with positive signal for very low IgE >0.10 kUA/L (E = allergen extract; M = recombinant molecular allergen).

| **POLLEN** | | | | | | | | | | | | | |
| --- | --- | --- | --- | --- | --- | --- | --- | --- | --- | --- | --- | --- | --- |
| **Category** | **Subcategory** | **Allergen** | **E/M** | **P_1** | **P_2** | **P_3** | **Category** | **Subcategory** | **Allergen** | **E/M** | **P_1** | **P_2** | **P_3** |
| Grass pollen | Bermuda grass | Cyn d | E | ≤0.10 | ≤0.10 | ≤0.10 | Tree pollen | Acacia | Aca m | E | ≤0.10 | ≤0.10 | ≤0.10 |
|  | Perennial ryegrass | Cyn d 1 | M | ≤0.10 | ≤0.10 | ≤0.10 |  | Tree of heaven | Ail a | E | ≤0.10 | 0.17 | 0.29 |
|  | Timothy grass | Lol p 1 | M | ≤0.10 | ≤0.10 | ≤0.10 |  | Alder | Aln g 1 | M | ≤0.10 | ≤0.10 | ≤0.10 |
|  | Bahia grass | Pas n | E | ≤0.10 | ≤0.10 | ≤0.10 |  |  | Aln g 4 | M | ≤0.10 | ≤0.10 | ≤0.10 |
|  | Timothy grass | Phl p 1 | M | 0.23 | 0.24 | 0.20 |  | Silver birch | Bet v 1 | M | ≤0.10 | ≤0.10 | ≤0.10 |
|  |  | Phl p 2 | M | ≤0.10 | ≤0.10 | ≤0.10 |  |  | Bet v 2 | M | ≤0.10 | ≤0.10 | ≤0.10 |
|  |  | Phl p 5.0101 | M | ≤0.10 | ≤0.10 | ≤0.10 |  |  | Bet v 6 | M | ≤0.10 | ≤0.10 | ≤0.10 |
|  |  | Phl p 6 | M | ≤0.10 | ≤0.10 | ≤0.10 |  | Paper mulberry | Bro pa | E | ≤0.10 | ≤0.10 | ≤0.10 |
|  |  | Phl p 7 | M | ≤0.10 | ≤0.10 | ≤0.10 |  | Hazel pollen | Cor a_pollen | E | ≤0.10 | ≤0.10 | ≤0.10 |
|  |  | Phl p 12 | M | ≤0.10 | ≤0.10 | ≤0.10 |  |  | Cor a 1.0103 | M | ≤0.10 | ≤0.10 | ≤0.10 |
|  | Common reed | Phr c | E | ≤0.10 | ≤0.10 | 0.16 |  | Sugi | Cry j 1 | M | ≤0.10 | ≤0.10 | 0.16 |
|  | Cultivated rye, pollen | Sec c_pollen | E | ≤0.10 | ≤0.10 | ≤0.10 |  | Cypress | Cup a 1 | M | ≤0.10 | ≤0.10 | 0.12 |
| Weed pollen | Common pigweed | Ama r | E | ≤0.10 | ≤0.10 | ≤0.10 |  |  | Cup s | E | ≤0.10 | 0.25 | 0.34 |
|  | Ragweed | Amb a | E | ≤0.10 | ≤0.10 | ≤0.10 |  | Beech | Fag s 1 | M | ≤0.10 | 0.10 | 0.13 |
|  |  | Amb a 1 | M | ≤0.10 | ≤0.10 | ≤0.10 |  | Ash | Fra e | E | ≤0.10 | ≤0.10 | ≤0.10 |
|  |  | Amb a 4 | M | ≤0.10 | ≤0.10 | ≤0.10 |  |  | Fra e 1 | M | ≤0.10 | ≤0.10 | ≤0.10 |
|  | Mugwort | Art v | E | ≤0.10 | ≤0.10 | ≤0.10 |  | Walnut pollen | Jug r_pollen | E | ≤0.10 | ≤0.10 | ≤0.10 |
|  |  | Art v 1 | M | ≤0.10 | ≤0.10 | ≤0.10 |  | Mountain cedar | Jun a | E | ≤0.10 | ≤0.10 | ≤0.10 |
|  |  | Art v 3 | M | ≤0.10 | ≤0.10 | ≤0.10 |  | Mulberry | Mor r | E | ≤0.10 | ≤0.10 | ≤0.10 |
|  | Hemp | Can s | E | ≤0.10 | 0.11 | ≤0.10 |  | Olive | Ole e 1 | M | ≤0.10 | ≤0.10 | ≤0.10 |
|  |  | Can s 3 | M | ≤0.10 | ≤0.10 | ≤0.10 |  |  | Ole e 9 | M | ≤0.10 | ≤0.10 | ≤0.10 |
|  | Lamb's quarter | Che a | E | ≤0.10 | ≤0.10 | ≤0.10 |  | Date palm | Pho d 2 | M | ≤0.10 | ≤0.10 | ≤0.10 |
|  |  | Che a 1 | M | ≤0.10 | ≤0.10 | ≤0.10 |  | London plane tree | Pla a 1 | M | ≤0.10 | 0.10 | 0.14 |
|  | Annual mercury | Mer a 1 | M | ≤0.10 | ≤0.10 | ≤0.10 |  |  | Pla a 2 | M | ≤0.10 | ≤0.10 | ≤0.10 |
|  | Wall pellitory | Par j | E | ≤0.10 | ≤0.10 | ≤0.10 |  |  | Pla a 3 | M | ≤0.10 | ≤0.10 | ≤0.10 |
|  |  | Par j 2 | M | ≤0.10 | ≤0.10 | ≤0.10 |  | Cottonwood | Pop n | E | ≤0.10 | 0.71 | 0.83 |
|  | Ribwort | Pla l | E | ≤0.10 | ≤0.10 | ≤0.10 |  | Elm | Ulm c | E | ≤0.10 | 0.23 | 0.34 |
|  |  | Pla l 1 | M | ≤0.10 | 0.14 | ≤0.10 |  |  |  |  |  |  |  |
|  | Russian thistle | Sal k | E | ≤0.10 | 0.17 | 0.16 |  |  |  |  |  |  |  |
|  |  | Sal k 1 | M | ≤0.10 | ≤0.10 | ≤0.10 |  |  |  |  |  |  |  |
|  | Nettle | Urt d | E | ≤0.10 | ≤0.10 | ≤0.10 |  |  |  |  |  |  |  |
| **INSECT/VENOM** | | | | | | | | | | | | | |
| **Category** | **Subcategory** | **Allergen** | **E/M** | **P_1** | **P_2** | **P_3** | **Category** | **Subcategory** | **Allergen** | **E/M** | **P_1** | **P_2** | **P_3** |
| Honey bee venom | Honey bee | Api m | E | ≤0.10 | ≤0.10 | ≤0.10 | Cockroach | German cockroach | Bla g 1 | M | ≤0.10 | ≤0.10 | ≤0.10 |
|  |  | Api m 1 | M | ≤0.10 | ≤0.10 | ≤0.10 |  |  | Bla g 2 | M | ≤0.10 | ≤0.10 | ≤0.10 |
|  |  | Api m 10 | M | ≤0.10 | ≤0.10 | ≤0.10 |  |  | Bla g 4 | M | ≤0.10 | ≤0.10 | ≤0.10 |
| Wasp venom | Hornet | Dol spp. | E | ≤0.10 | ≤0.10 | ≤0.10 |  |  | Bla g 5 | M | ≤0.10 | ≤0.10 | ≤0.10 |
|  | Paper wasp venom | Pol d | E | ≤0.10 | 0.22 | 0.18 |  |  | Bla g 9 | M | ≤0.10 | ≤0.10 | ≤0.10 |
|  |  | Pol d 5 | M | ≤0.10 | ≤0.10 | ≤0.10 |  | American cockroach | Per a | E | ≤0.10 | ≤0.10 | ≤0.10 |
|  | Wasp venom | Ves v | E | ≤0.10 | 0.13 | ≤0.10 |  |  | Per a 7 | M | ≤0.10 | ≤0.10 | ≤0.10 |
|  |  | Ves v 1 | M | ≤0.10 | ≤0.10 | ≤0.10 | 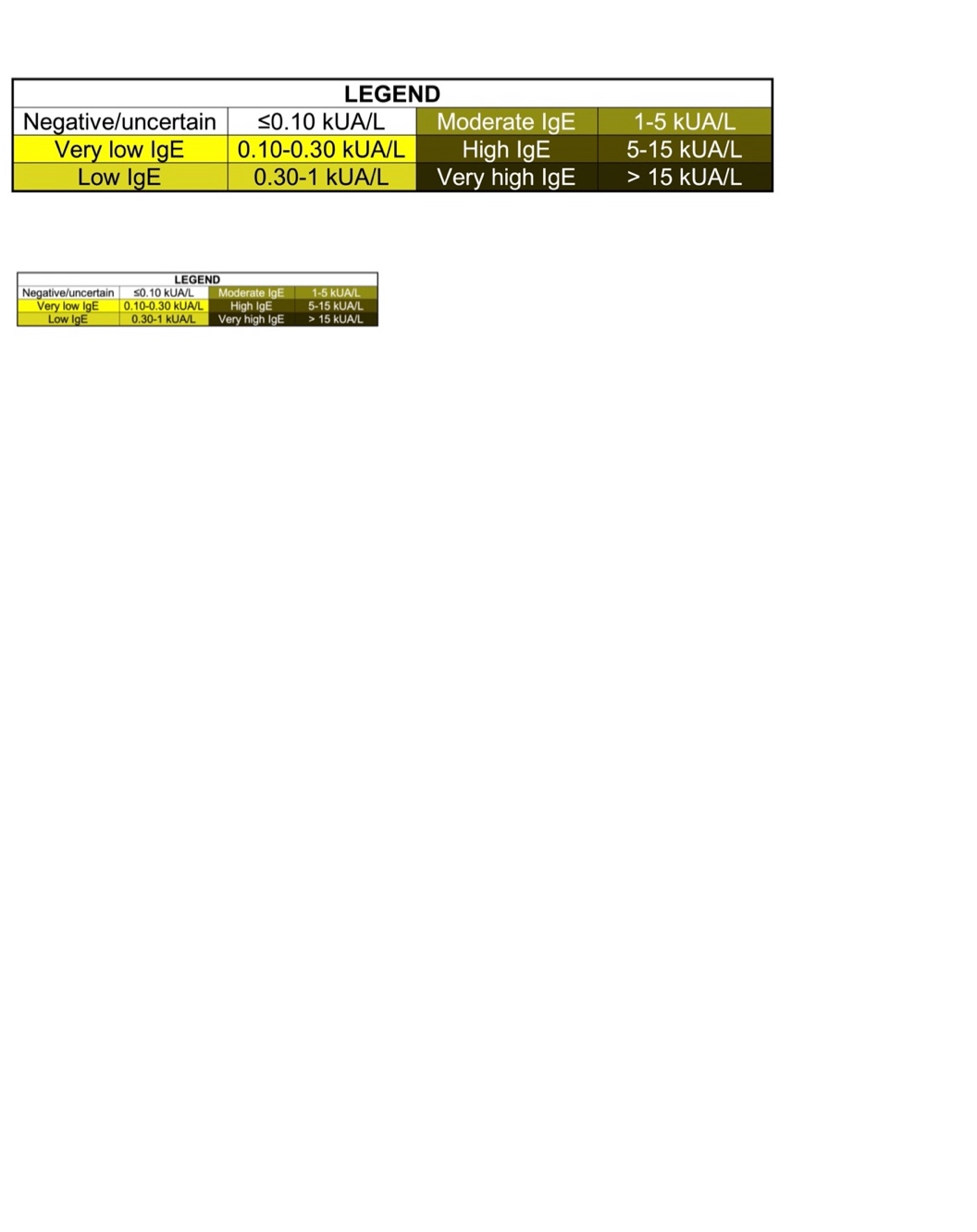 | | | | | | |
|  |  | Ves v 5 | M | ≤0.10 | ≤0.10 | ≤0.10 |  |  |  |  |  |  |  |
| Fire ant poison | Fire ant | Sol spp. | E | ≤0.10 | ≤0.10 | ≤0.10 |  |  |  |  |  |  |  |
|  | | | | | | | | | | | | | |
| **MITE** | | | | | | | | | | | | | |
| **Category** | **Subcategory** | **Allergen** | **E/M** | **P_1** | **P_2** | **P_3** | **Category** | **Subcategory** | **Allergen** | **E/M** | **P_1** | **P_2** | **P_3** |
| House dust mite | American house dust mite | Der f 1 | M | ≤0.10 | ≤0.10 | ≤0.10 | Storage mite | Acarus siro | Aca s | E | ≤0.10 | ≤0.10 | ≤0.10 |
|  |  | Der f 2 | M | ≤0.10 | ≤0.10 | ≤0.10 |  | Blomia tropicalis | Blo t 5 | M | ≤0.10 | 0.17 | ≤0.10 |
|  | European house dust mite | Der p 1 | M | ≤0.10 | ≤0.10 | ≤0.10 |  |  | Blo t 10 | M | 0.10 | ≤0.10 | ≤0.10 |
|  |  | Der p 2 | M | ≤0.10 | ≤0.10 | ≤0.10 |  |  | Blo t 21 | M | ≤0.10 | ≤0.10 | ≤0.10 |
|  |  | Der p 5 | M | ≤0.10 | ≤0.10 | ≤0.10 |  | Glycyphagus domesticus | Gly d 2 | M | ≤0.10 | 0.18 | 0.14 |
|  |  | Der p 7 | M | ≤0.10 | 0.15 | ≤0.10 |  | Lepidoglyphus destructor | Lep d 2 | M | ≤0.10 | ≤0.10 | ≤0.10 |
|  |  | Der p 10 | M | ≤0.10 | ≤0.10 | ≤0.10 |  | Tyrophagus putrescentiae | Tyr p | E | ≤0.10 | ≤0.10 | ≤0.10 |
|  |  | Der p 11 | M | ≤0.10 | ≤0.10 | ≤0.10 |  |  | Tyr p 2 | M | ≤0.10 | ≤0.10 | ≤0.10 |
|  |  | Der p 20 | M | ≤0.10 | ≤0.10 | ≤0.10 |  |  |  |  |  |  |  |
|  |  | Der p 21 | M | ≤0.10 | 0.11 | ≤0.10 |  |  |  |  |  |  |  |
|  |  | Der p 23 | M | ≤0.10 | ≤0.10 | ≤0.10 |  |  |  |  |  |  |  |
| **ANIMAL ORIGIN** | | | | | | | **MICROORGANISM/SPORE** | | | | | | |
| **Category** | **Subcategory** | **Allergen** | **E/M** | **P_1** | **P_2** | **P_3** | **Category** | **Subcategory** | **Allergen** | **E/M** | **P_1** | **P_2** | **P_3** |
| Pet | Dog | Can f_Fd1 | M | ≤0.10 | ≤0.10 | ≤0.10 | Yeast | Malassezia sympodialis | Mala s 5 | M | ≤0.10 | ≤0.10 | ≤0.10 |
|  |  | Can f 1 | M | ≤0.10 | ≤0.10 | ≤0.10 |  |  | Mala s 6 | M | ≤0.10 | ≤0.10 | ≤0.10 |
|  |  | Can f 2 | M | ≤0.10 | ≤0.10 | ≤0.10 |  |  | Mala s 11 | M | ≤0.10 | ≤0.10 | ≤0.10 |
|  |  | Can f 3 | M | ≤0.10 | ≤0.10 | ≤0.10 |  | Yeast | Sac c | E | 0.15 | 0.10 | 0.11 |
|  |  | Can f 4 | M | ≤0.10 | ≤0.10 | ≤0.10 | Mold | Alternaria alternata | Alt a 1 | M | ≤0.10 | ≤0.10 | ≤0.10 |
|  |  | Can f 6 | M | ≤0.10 | ≤0.10 | ≤0.10 |  |  | Alt a 6 | M | ≤0.10 | ≤0.10 | ≤0.10 |
|  | Male dog urine (incl. Can f 5) | Can f 1_male urine | E | ≤0.10 | ≤0.10 | ≤0.10 |  | Aspergillus fumigatus | Asp f 1 | M | ≤0.10 | ≤0.10 | ≤0.10 |
|  | Guinea pig | Cav p 1 | M | ≤0.10 | ≤0.10 | ≤0.10 |  |  | Asp f 3 | M | ≤0.10 | ≤0.10 | ≤0.10 |
|  | Cat | Fel d 1 | M | ≤0.10 | ≤0.10 | ≤0.10 |  |  | Asp f 4 | M | ≤0.10 | ≤0.10 | ≤0.10 |
|  |  | Fel d 2 | M | ≤0.10 | ≤0.10 | ≤0.10 |  |  | Asp f 6 | M | ≤0.10 | ≤0.10 | ≤0.10 |
|  |  | Fel d 4 | M | ≤0.10 | ≤0.10 | ≤0.10 |  | Cladosporium herbarum | Cla h | E | ≤0.10 | ≤0.10 | ≤0.10 |
|  |  | Fel d 7 | M | ≤0.10 | ≤0.10 | ≤0.10 |  |  | Cla h 8 | M | ≤0.10 | ≤0.10 | ≤0.10 |
|  | House mouse | Mus m 1 | M | ≤0.10 | ≤0.10 | ≤0.10 |  | Penicillum chrysogenum | Pen ch | E | ≤0.10 | ≤0.10 | ≤0.10 |
|  | Rabbit, epithelium | Ory c 1 | M | ≤0.10 | ≤0.10 | ≤0.10 | **OTHERS** | | | | | | |
|  |  | Ory c 2 | M | ≤0.10 | ≤0.10 | ≤0.10 | **Category** | **Subcategory** | **Allergen** | **E/M** | **P_1** | **P_2** | **P_3** |
|  |  | Ory c 3 | M | ≤0.10 | ≤0.10 | ≤0.10 | Latex | | Hev b 1 | M | ≤0.10 | ≤0.10 | ≤0.10 |
|  | Djungarian hamster | Phod s 1 | M | ≤0.10 | ≤0.10 | ≤0.10 |  |  | Hev b 3 | M | ≤0.10 | ≤0.10 | 0.10 |
|  | Rat | Rat n | E | ≤0.10 | ≤0.10 | ≤0.10 |  |  | Hev b 5 | M | ≤0.10 | ≤0.10 | ≤0.10 |
| Farm animal | Cattle | Bos d 2 | M | ≤0.10 | ≤0.10 | ≤0.10 |  |  | Hev b 6.02 | M | ≤0.10 | ≤0.10 | ≤0.10 |
|  | Goat, epithelium | Cap h_epi. | E | ≤0.10 | ≤0.10 | ≤0.10 |  |  | Hev b 8 | M | ≤0.10 | ≤0.10 | ≤0.10 |
|  | Horse, epithelium | Equ c 1 | M | ≤0.10 | ≤0.10 | ≤0.10 |  |  | Hev b 11 | M | ≤0.10 | ≤0.10 | ≤0.10 |
|  |  | Equ c 3 | M | ≤0.10 | ≤0.10 | ≤0.10 | Ficus | Weeping fig | Fic b | E | 0.10 | 0.23 | ≤0.10 |
|  |  | Equ c 4 | M | ≤0.10 | ≤0.10 | ≤0.10 | CCD | Hom's lactoferrin | Hom s LF | M | ≤0.10 | ≤0.10 | ≤0.10 |
|  | Sheep, epithelium | Ovi a_epi. | E | ≤0.10 | ≤0.10 | ≤0.10 | Parasite | Pigeon tick | Arg r 1 | M | ≤0.10 | ≤0.10 | ≤0.10 |
|  | Pig | Sus d_epi. | E | ≤0.10 | ≤0.10 | ≤0.10 |  |  |  |  |  |  |  |
|  |  |  |  |  |  |  | 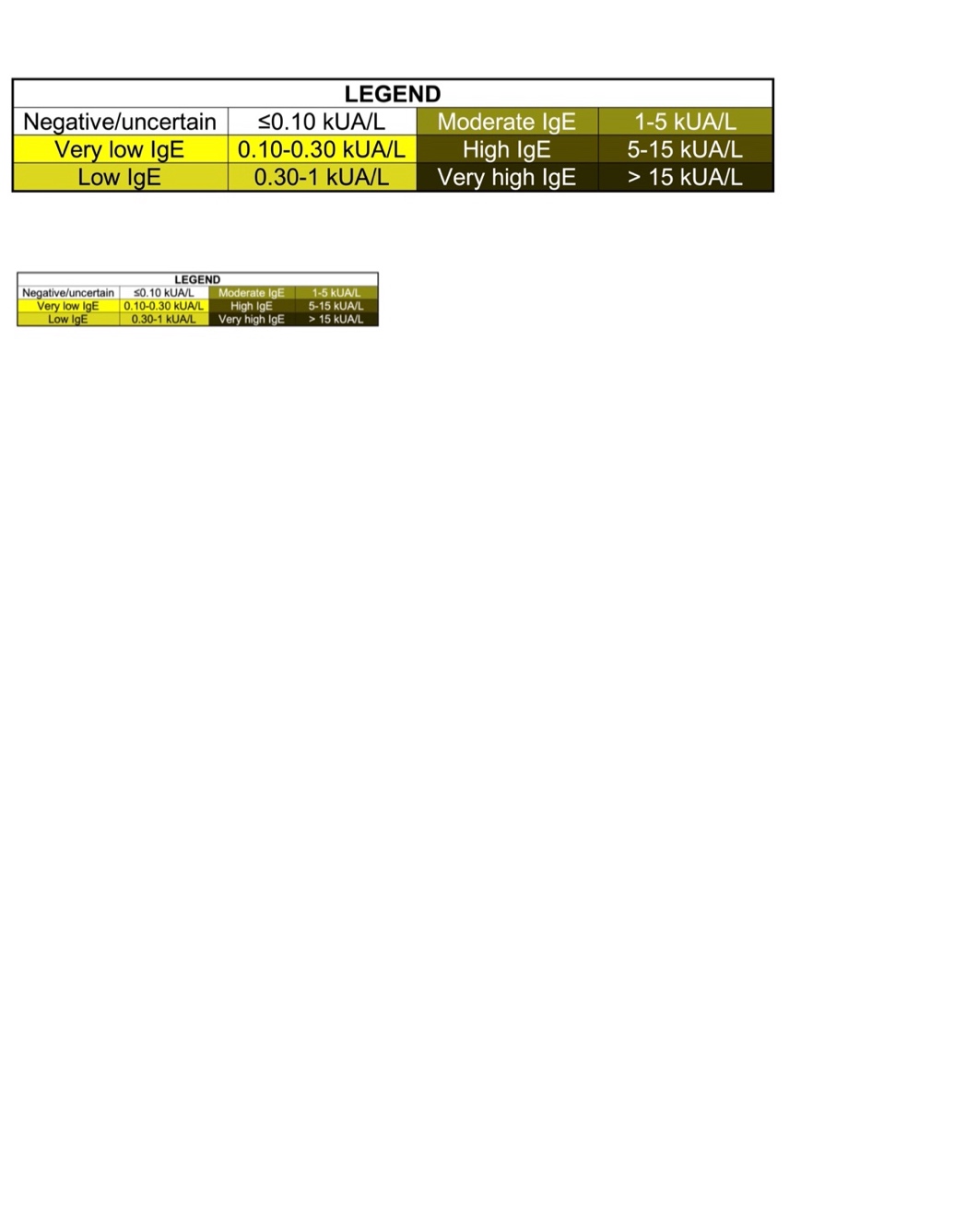 | | | | | | |
|  |  |  |  |  |  |  |  |  |  |  |  |  |  |
|  |  |  |  |  |  |  |  |  |  |  |  |  |  |
| **PLANT FOOD** | | | | | | | | | | | | | |
| **Category** | **Subcategory** | **Allergen** | **E/M** | **P_1** | **P_2** | **P_3** | **Category** | **Subcategory** | **Allergen** | **E/M** | **P_1** | **P_2** | **P_3** |
| Legume | Peanut | Ara h 1 | M | ≤0.10 | ≤0.10 | 0.12 | Cereal | Oat | Ave s | E | ≤0.10 | 0.17 | 0.15 |
|  |  | Ara h 2 | M | ≤0.10 | ≤0.10 | ≤0.10 |  | Quinoa | Che q | E | ≤0.10 | ≤0.10 | 0.16 |
|  |  | Ara h 3 | M | ≤0.10 | ≤0.10 | ≤0.10 |  | Common buckwheat | Fag e | E | ≤0.10 | 0.20 | 0.25 |
|  |  | Ara h 6 | M | ≤0.10 | ≤0.10 | ≤0.10 |  |  | Fag e 2 | M | ≤0.10 | ≤0.10 | ≤0.10 |
|  |  | Ara h 8 | M | ≤0.10 | 0.12 | 0.16 |  | Barley | Hor v | E | ≤0.10 | 0.21 | 0.16 |
|  |  | Ara h 9 | M | ≤0.10 | ≤0.10 | ≤0.10 |  | Lupine seed | Lup a | E | ≤0.10 | 0.15 | 0.21 |
|  |  | Ara h 15 | M | ≤0.10 | ≤0.10 | ≤0.10 |  | Rice | Ory s | E | 0.21 | 0.28 | 0.16 |
|  | Chickpea | Cic a | E | ≤0.10 | 0.12 | 0.18 |  | Millet | Pan m | E | 0.17 | 1.04 | 1.35 |
|  | Soy | Gly m 4 | M | ≤0.10 | 0.11 | ≤0.10 |  | Cultivated rye | Sec c_flour | E | ≤0.10 | 0.15 | 0.22 |
|  |  | Gly m 5 | M | ≤0.10 | ≤0.10 | ≤0.10 |  | Wheat | Tri a aA_TI | M | ≤0.10 | ≤0.10 | ≤0.10 |
|  |  | Gly m 6 | M | ≤0.10 | ≤0.10 | ≤0.10 |  |  | Tri a 14 | M | ≤0.10 | ≤0.10 | ≤0.10 |
|  |  | Gly m 8 | M | ≤0.10 | ≤0.10 | ≤0.10 |  |  | Tri a 19 | M | 0.11 | 0.13 | 0.21 |
|  | Lentil | Len c | E | ≤0.10 | ≤0.10 | 0.14 |  | Spelt | Tri s | E | ≤0.10 | 0.10 | ≤0.10 |
|  | White bean | Pha v | E | ≤0.10 | ≤0.10 | ≤0.10 |  | Maize | Zea m | E | ≤0.10 | ≤0.10 | ≤0.10 |
|  | Pea | Pis s | E | ≤0.10 | 0.10 | ≤0.10 |  |  | Zea m 14 | M | ≤0.10 | ≤0.10 | ≤0.10 |
| Nut | Cashew | Ana o | E | ≤0.10 | 0.26 | 0.22 | Fruit | Kiwi | Act d 1 | M | ≤0.10 | 0.28 | 0.40 |
|  |  | Ana o 2 | M | ≤0.10 | ≤0.10 | ≤0.10 |  |  | Act d 2 | M | ≤0.10 | 0.22 | 0.19 |
|  |  | Ana o 3 | M | ≤0.10 | ≤0.10 | ≤0.10 |  |  | Act d 5 | M | ≤0.10 | ≤0.10 | ≤0.10 |
|  | Brazil nut | Ber e | E | ≤0.10 | ≤0.10 | ≤0.10 |  |  | Act d 10 | M | ≤0.10 | ≤0.10 | ≤0.10 |
|  |  | Ber e 1 | M | ≤0.10 | ≤0.10 | ≤0.10 |  | Papaya | Car p | E | ≤0.10 | ≤0.10 | ≤0.10 |
|  | Pecan | Car i | E | ≤0.10 | 0.18 | 0.24 |  | Orange | Cit s | E | ≤0.10 | ≤0.10 | ≤0.10 |
|  | Hazelnut | Cor a 1.0401 | M | ≤0.10 | ≤0.10 | ≤0.10 |  | Melon | Cuc m 2 | M | ≤0.10 | ≤0.10 | ≤0.10 |
|  |  | Cor a 8 | M | ≤0.10 | ≤0.10 | ≤0.10 |  | Fig | Fic c | E | ≤0.10 | ≤0.10 | ≤0.10 |
|  |  | Cor a 9 | M | ≤0.10 | ≤0.10 | ≤0.10 |  | Strawberry | Fra a 1+3 | M | ≤0.10 | ≤0.10 | ≤0.10 |
|  |  | Cor a 11 | M | ≤0.10 | ≤0.10 | 0.11 |  | Apple | Mal d 1 | M | ≤0.10 | ≤0.10 | ≤0.10 |
|  |  | Cor a 14 | M | ≤0.10 | ≤0.10 | ≤0.10 |  |  | Mal d 2 | M | ≤0.10 | ≤0.10 | ≤0.10 |
|  | Walnut | Jug r 1 | M | ≤0.10 | 0.17 | 0.23 |  |  | Mal d 3 | M | ≤0.10 | ≤0.10 | ≤0.10 |
|  |  | Jug r 2 | M | ≤0.10 | ≤0.10 | ≤0.10 |  | Mango | Man i | E | ≤0.10 | ≤0.10 | ≤0.10 |
|  |  | Jug r 3 | M | ≤0.10 | ≤0.10 | ≤0.10 |  | Banana | Mus a | E | ≤0.10 | ≤0.10 | ≤0.10 |
|  |  | Jug r 4 | M | ≤0.10 | ≤0.10 | ≤0.10 |  | Avocado | Pers a | E | ≤0.10 | ≤0.10 | ≤0.10 |
|  |  | Jug r 6 | M | ≤0.10 | ≤0.10 | 0.17 |  | Cherry | Pru av | E | ≤0.10 | ≤0.10 | ≤0.10 |
|  | Macadamia | Mac i 2S Alb | M | ≤0.10 | ≤0.10 | ≤0.10 |  | Peach | Pru p 3 | M | ≤0.10 | ≤0.10 | ≤0.10 |
|  |  | Mac inte | E | ≤0.10 | 0.10 | 0.16 |  | Pear | Pyr c | E | ≤0.10 | ≤0.10 | ≤0.10 |
|  | Pistachio | Pis v 1 | M | ≤0.10 | ≤0.10 | ≤0.10 |  | Blueberry | Vac m | E | ≤0.10 | ≤0.10 | ≤0.10 |
|  |  | Pis v 2 | M | ≤0.10 | ≤0.10 | ≤0.10 |  | Grape | Vit v 1 | M | ≤0.10 | ≤0.10 | ≤0.10 |
|  |  | Pis v 3 | M | ≤0.10 | ≤0.10 | ≤0.10 | Vegetable | Onion | All c | E | ≤0.10 | 0.18 | 0.21 |
|  | Almond | Pru du | E | ≤0.10 | ≤0.10 | ≤0.10 |  | Garlic | All s | E | ≤0.10 | ≤0.10 | 0.13 |
| Seed | Pumpkin seed | Cuc p | E | ≤0.10 | 0.34 | 0.86 |  | Celery | Api g 1 | M | ≤0.10 | ≤0.10 | ≤0.10 |
|  | Sunflower s. | Hel a | E | ≤0.10 | ≤0.10 | ≤0.10 |  |  | Api g 2 | M | ≤0.10 | ≤0.10 | ≤0.10 |
|  | Poppy seed | Pap s | E | ≤0.10 | ≤0.10 | ≤0.10 |  |  | Api g 6 | M | ≤0.10 | ≤0.10 | ≤0.10 |
|  |  | Pap s 2S Alb | M | ≤0.10 | ≤0.10 | ≤0.10 |  | Carrot | Dau c | E | ≤0.10 | ≤0.10 | ≤0.10 |
|  | Sesame | Ses i | E | ≤0.10 | ≤0.10 | ≤0.10 |  |  | Dau c 1 | M | ≤0.10 | ≤0.10 | ≤0.10 |
|  |  | Ses i 1 | M | ≤0.10 | ≤0.10 | ≤0.10 |  | Potato | Sol t | E | ≤0.10 | 0.12 | 0.19 |
|  | Fenugreek s. | Tri fo | E | ≤0.10 | ≤0.10 | ≤0.10 |  | Tomato | Sola l | E | ≤0.10 | ≤0.10 | ≤0.10 |
| Spice | Paprika | Cap a | E | ≤0.10 | 0.34 | 0.48 |  |  | Sola l 6 | M | ≤0.10 | ≤0.10 | ≤0.10 |
|  | Caraway | Car c | E | ≤0.10 | ≤0.10 | ≤0.10 |  |  |  |  |  |  |  |
|  | Oregano | Ori v | E | ≤0.10 | ≤0.10 | ≤0.10 |  |  |  |  |  |  |  |
|  | Parsley | Pet c | E | ≤0.10 | ≤0.10 | 0.15 | 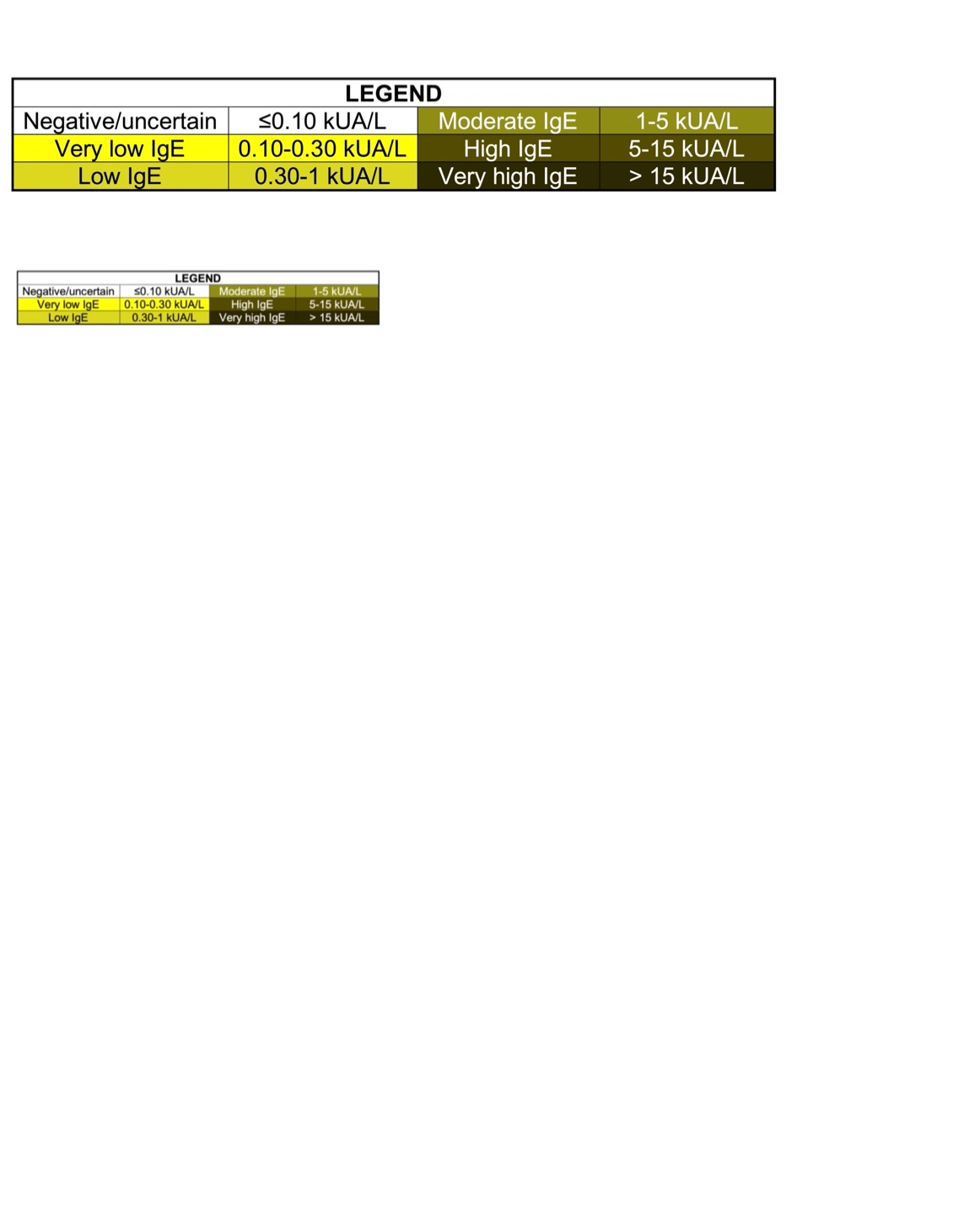 | | | | | | |
|  | Anise | Pim a | E | ≤0.10 | ≤0.10 | ≤0.10 |  |  |  |  |  |  |  |
|  | Mustard | Sin / Sin a 1 | E/M | ≤0.10 | ≤0.10 | ≤0.10 |  |  |  |  |  |  |  |
| **ANIMAL FOOD** | | | | | | | | | | | | | |
| **Category** | **Subcategory** | **Allergen** | **E/M** | **P_1** | **P_2** | **P_3** | **Category** | **Subcategory** | **Allergen** | **E/M** | **P_1** | **P_2** | **P_3** |
| Seafood | Herring worm | Ani s 1 | M | 0.18 | 0.16 | ≤0.10 | Milk | Cow, milk | Bos d_milk | E | ≤0.10 | ≤0.10 | ≤0.10 |
|  |  | Ani s 3 | M | ≤0.10 | ≤0.10 | ≤0.10 |  |  | Bos d 4 | M | ≤0.10 | ≤0.10 | ≤0.10 |
|  | Crab | Chi spp. | E | ≤0.10 | ≤0.10 | ≤0.10 |  |  | Bos d 5 | M | ≤0.10 | ≤0.10 | ≤0.10 |
|  | Herring | Clu h | E | ≤0.10 | ≤0.10 | ≤0.10 |  |  | Bos d 8 | M | ≤0.10 | ≤0.10 | ≤0.10 |
|  |  | Clu h 1 | M | ≤0.10 | ≤0.10 | ≤0.10 |  | Camel, milk | Cam d | E | ≤0.10 | 0.36 | 0.34 |
|  | Brown shrimp | Cra c 6 | M | ≤0.10 | ≤0.10 | ≤0.10 |  | Goat, milk | Cap h_milk | E | ≤0.10 | 0.10 | ≤0.10 |
|  | Carp | Cyp c 1 | M | ≤0.10 | ≤0.10 | ≤0.10 |  | Mare's milk | Equ c_milk | E | ≤0.10 | ≤0.10 | ≤0.10 |
|  | Atlantic cod | Gad m | E | ≤0.10 | ≤0.10 | ≤0.10 |  | Sheep, milk | Ovi a_milk | E | ≤0.10 | ≤0.10 | ≤0.10 |
|  |  | Gad m 2+3 | M | ≤0.10 | ≤0.10 | ≤0.10 | Egg | Egg white | Gal d_white | E | 0.12 | 0.12 | 0.14 |
|  |  | Gad m 1 | M | ≤0.10 | ≤0.10 | ≤0.10 |  |  | Gal d 1 | M | ≤0.10 | ≤0.10 | ≤0.10 |
|  | Lobster | Hom g | E | ≤0.10 | 0.10 | ≤0.10 |  |  | Gal d 2 | M | ≤0.10 | ≤0.10 | ≤0.10 |
|  | Shrimp | Lit s | E | ≤0.10 | ≤0.10 | ≤0.10 |  |  | Gal d 3 | M | ≤0.10 | ≤0.10 | ≤0.10 |
|  | Squid | Lol spp. | E | ≤0.10 | ≤0.10 | ≤0.10 |  |  | Gal d 4 | M | ≤0.10 | ≤0.10 | ≤0.10 |
|  | Common mussel | Myt e | E | ≤0.10 | ≤0.10 | ≤0.10 |  | Egg yolk | Gal d_yolk | E | 0.13 | 0.19 | 0.22 |
|  | Oyster | Ost e | E | ≤0.10 | ≤0.10 | ≤0.10 |  |  | Gal d 5 | M | ≤0.10 | ≤0.10 | ≤0.10 |
|  | Shrimp | Pan b | E | ≤0.10 | ≤0.10 | ≤0.10 | Meat | House cricket | Ach d | E | ≤0.10 | 0.14 | 0.13 |
|  | Scallop | Pec spp. | E | ≤0.10 | ≤0.10 | ≤0.10 |  | Cattle, meat | Bos d_meat | E | ≤0.10 | ≤0.10 | ≤0.10 |
|  | Black tiger shrimp | Pen m 1 | M | ≤0.10 | ≤0.10 | ≤0.10 |  |  | Bos d 6 | M | ≤0.10 | ≤0.10 | ≤0.10 |
|  |  | Pen m 2 | M | ≤0.10 | ≤0.10 | ≤0.10 |  | Horse, meat | Equ c_meat | E | ≤0.10 | ≤0.10 | ≤0.10 |
|  |  | Pen m 3 | M | ≤0.10 | ≤0.10 | 0.10 |  | Chicken, meat | Gal d_meat | E | ≤0.10 | ≤0.10 | ≤0.10 |
|  |  | Pen m 4 | M | ≤0.10 | ≤0.10 | ≤0.10 |  | Migratory locust | Loc m | E | ≤0.10 | ≤0.10 | ≤0.10 |
|  | Thornback ray | Raj c | E | ≤0.10 | ≤0.10 | ≤0.10 |  | Turkey | Mel g | E | ≤0.10 | 0.10 | ≤0.10 |
|  |  | Raj c Parvalbumin | M | ≤0.10 | ≤0.10 | ≤0.10 |  | Rabbit, meat | Ory_meat | E | ≤0.10 | ≤0.10 | 0.16 |
|  | Clam | Rud spp. | E | ≤0.10 | ≤0.10 | ≤0.10 |  | Sheep, meat | Ovi a_meat | E | ≤0.10 | ≤0.10 | ≤0.10 |
|  | Salmon | Sal s | E | ≤0.10 | ≤0.10 | ≤0.10 |  | Pork | Sus d_meat | E | ≤0.10 | ≤0.10 | ≤0.10 |
|  |  | Sal s 1 | M | ≤0.10 | ≤0.10 | ≤0.10 |  |  | Sus d 1 | M | ≤0.10 | ≤0.10 | ≤0.10 |
|  | Atlantic mackerel | Sco s | E | ≤0.10 | ≤0.10 | ≤0.10 |  | Mealworm | Ten m | E | ≤0.10 | ≤0.10 | ≤0.10 |
|  |  | Sco s 1 | M | ≤0.10 | ≤0.10 | ≤0.10 | 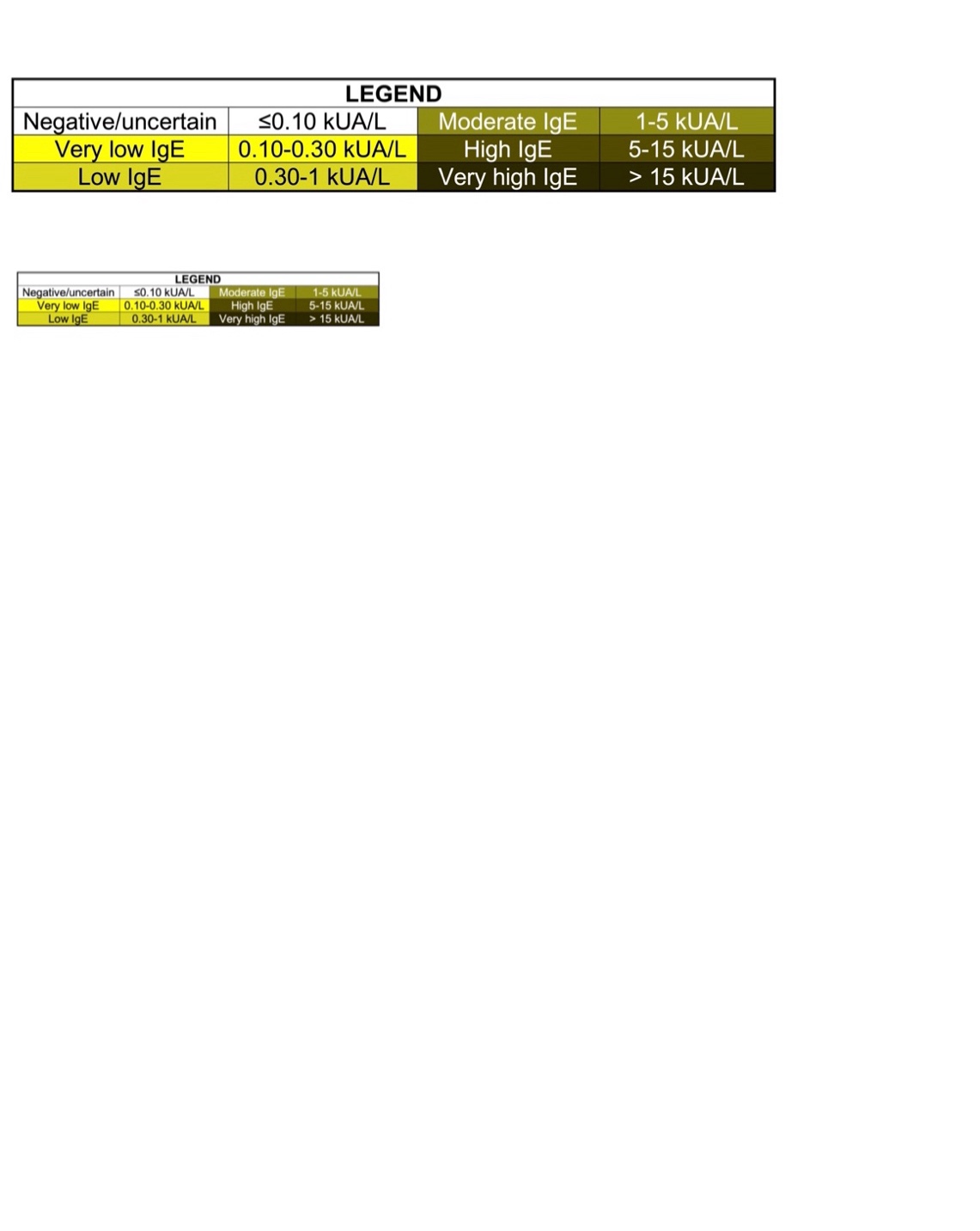 | | | | | | |
|  | Tuna | Thu a | E | ≤0.10 | ≤0.10 | ≤0.10 |  |  |  |  |  |  |  |
|  |  | Thu a 1 | M | ≤0.10 | ≤0.10 | ≤0.10 |  |  |  |  |  |  |  |
|  | Swordfish | Xip g 1 | M | ≤0.10 | ≤0.10 | ≤0.10 |  |  |  |  |  |  |  |

1. **Supplementary Figure S1**

**Supplementary Figure S1: Flow cytometry gating strategy for human plasma cells and memory B cells.** Gating strategy for human plasma cells (PC) and memory B cells (MBC) is shown using stained cells from an engrafted NSG-SGM3 mouse cultured *ex vivo* for 96 hours with anti-human CD40 (anti-hCD40; CD40L) and human IL-4 Th2 cytokines. Human PC were sequentially gated on singlet, live, human CD138^+^ human CD19^var^, human IgD^-^, human CD23^-^, human CD138^+^ human CD27^+^ cells. Human MBC were gated sequentially on singlet, live, human CD19^+^ human CD138^-^, human IgD^-^, human CD23^-^, human CD20^+^ human CD27^+/hi^ cells. This approach thus excludes follicular, marginal zone, and germinal center B cells that may otherwise also express positive signal from the final gate.


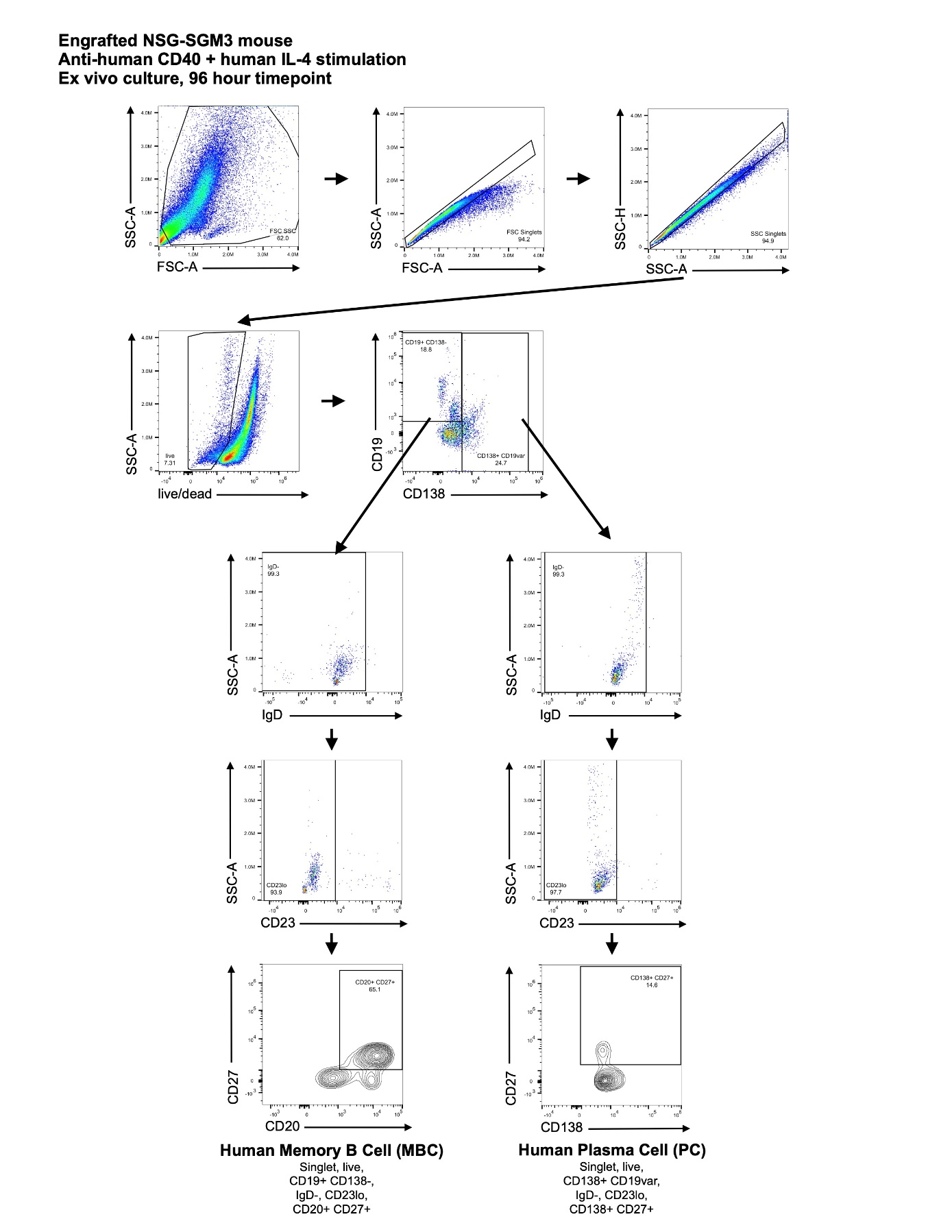


1. **Supplementary Figure S2**

**Supplementary Figure S2: Flow cytometry gating strategy for murine plasma cells / plasmablasts and memory B cells.**

Gating strategy for murine plasma cells (PC) and memory B cells (MBC) is shown using stained cells from a wildtype B6 mouse cultured *ex vivo* for 96 hours with anti-mouse CD40 (anti-mCD40/CD40L) and murine IL-4 stimulatory cytokines. Murine PC were gated sequentially on singlet, live, mouse CD138^hi^, mouse IgD^-^ mouse CD22^-^, mouse/human GL7^lo^, mouse/human B220^lo^ cells; thereafter, mouse PC were gated as mouse CD138^+^ mouse CD19^+^ and mouse PB were gated as mouse CD138^+^ mouse CD19^-^. Murine MBC were gated sequentially on singlet, live, mouse CD138^-^, mouse IgD^-^ mouse CD22^-^, mouse/human GL7^lo^, mouse CD273^+^ mouse/human B220^+^ cells. This approach thus excludes follicular, marginal zone, and germinal center B cells that may otherwise also express positive signal from the final gate.


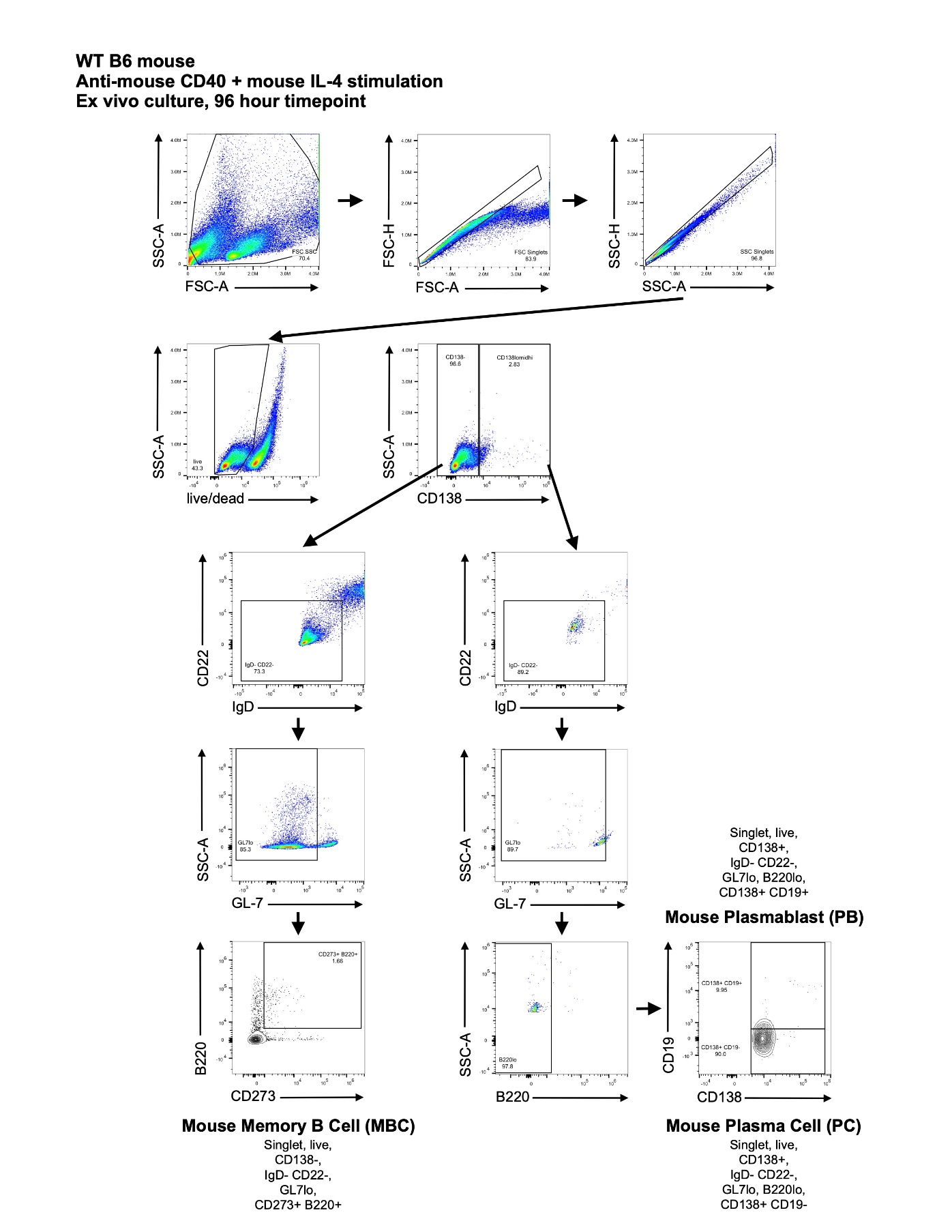


1. **Supplementary Figure S3**

**Supplementary Figure S3: Anti-human and anti-murine flow cytometry antibodies used for plasma cell and memory B cell identification are minimally cross-reactive. (A)** Histograms of fluorescent signal in seven individual fluorophore channels for various donor human or murine blood samples stained with fluorescently-conjugated anti-human antibodies. Cells were gated on singlets for unstained human blood (light purple) and single color-stained human blood (orange) samples, or on singlet live cells for full panel-stained murine blood from WT (gray), non-engrafted NSG-SGM3 (light blue), and engrafted NSG-SGM3 (red) mice. Y-axis values indicate modal count (percentage of maximum count). **(B)** Histograms of fluorescent signal in seven individual fluorophore channels for various donor human or murine blood samples stained with fluorescently-conjugated anti-mouse antibodies. Cells were gated on singlets for unstained WT mouse blood (light purple) and single color-stained WT mouse blood (orange), or on singlet live cells for full panel-stained human blood (dark purple) or murine blood from WT (gray), non-engrafted NSG-SGM3 (light blue), and engrafted NSG-SGM3 (red) mice. Y-axis values indicate modal count (percentage of maximum count).


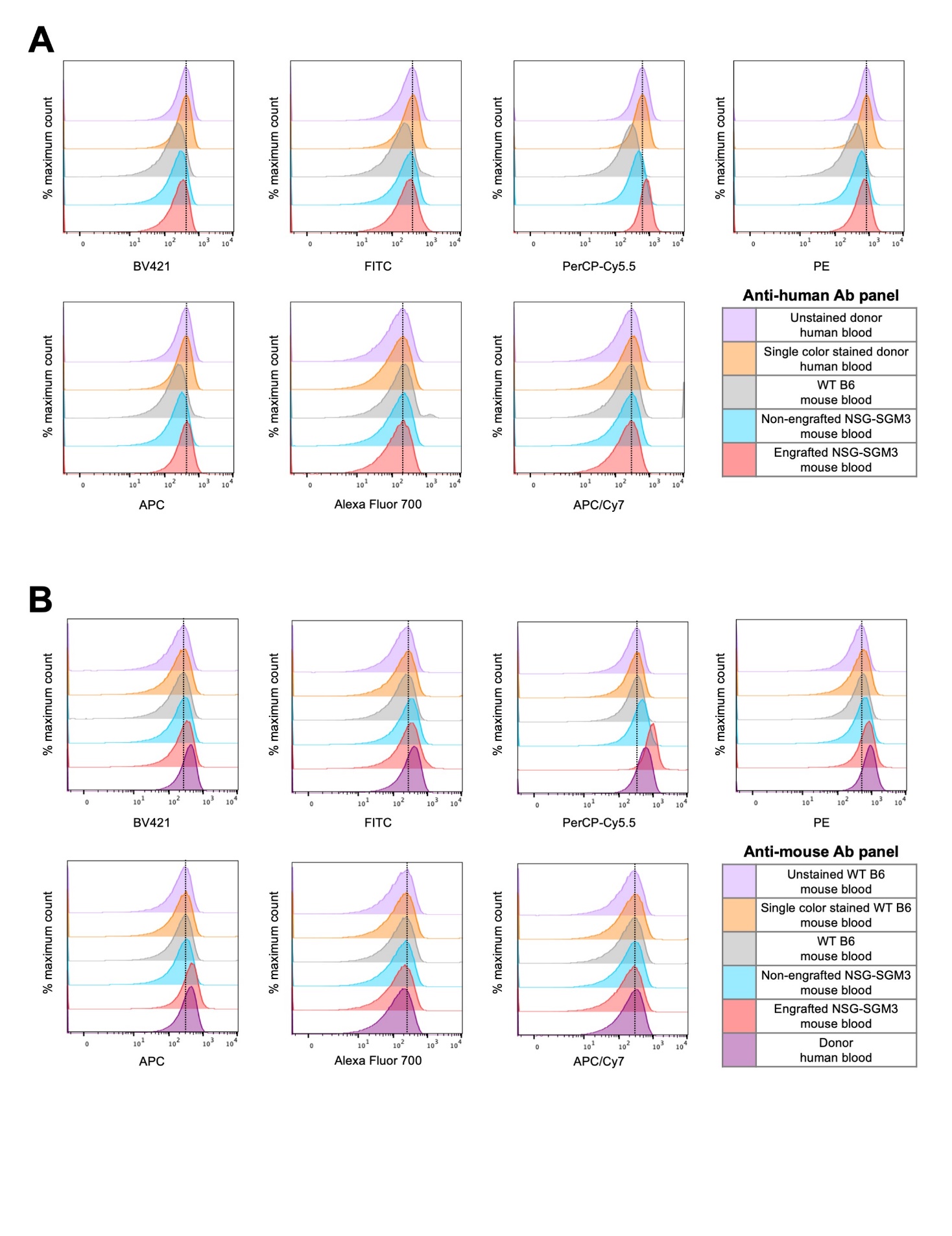


1. **Supplementary Figure S4**

**Supplementary Figure S4: Non-engrafted NSG-SGM3 splenocytes are poor controls for flow cytometry due to significant baseline autofluorescence. (A)** Baseline percentage of human PC, human MBC, murine PC/PB, and murine MBC from non-engrafted NSG-SGM3 splenocytes at zero hours prior to *ex vivo* culture. Values were calculated using cell counts of the population of interest, divided by all live cells (*n* = 4 mice/group, compiled from two independent experiments). **(B)** Histograms of baseline autofluorescent signal in eight individual fluorophore channels from unstained WT (gray), non-engrafted NSG-SGM3 (light blue), or engrafted NSG-SGM3 (red) splenocyte samples after 96 hours of *ex vivo* culture with human cytokines. Cells were gated on singlets. Y-axis values indicate modal count (percentage of maximum count). **(C)** Percentage of human PC, human MBC, murine PC/PB, and murine MBC from non-engrafted NSG-SGM3 splenocytes cultured *ex vivo* for 96 hours with either human or murine cytokines. Values were calculated using cell counts of the sequentially gated population of interest (**Fig. S1 and S2**), divided by all live cells (*n* = 6 mice/group, compiled from two independent experiments). Error bars in **A** and **C** represent SEM. P values < 0.05 were considered significant (unlabeled, not significant).


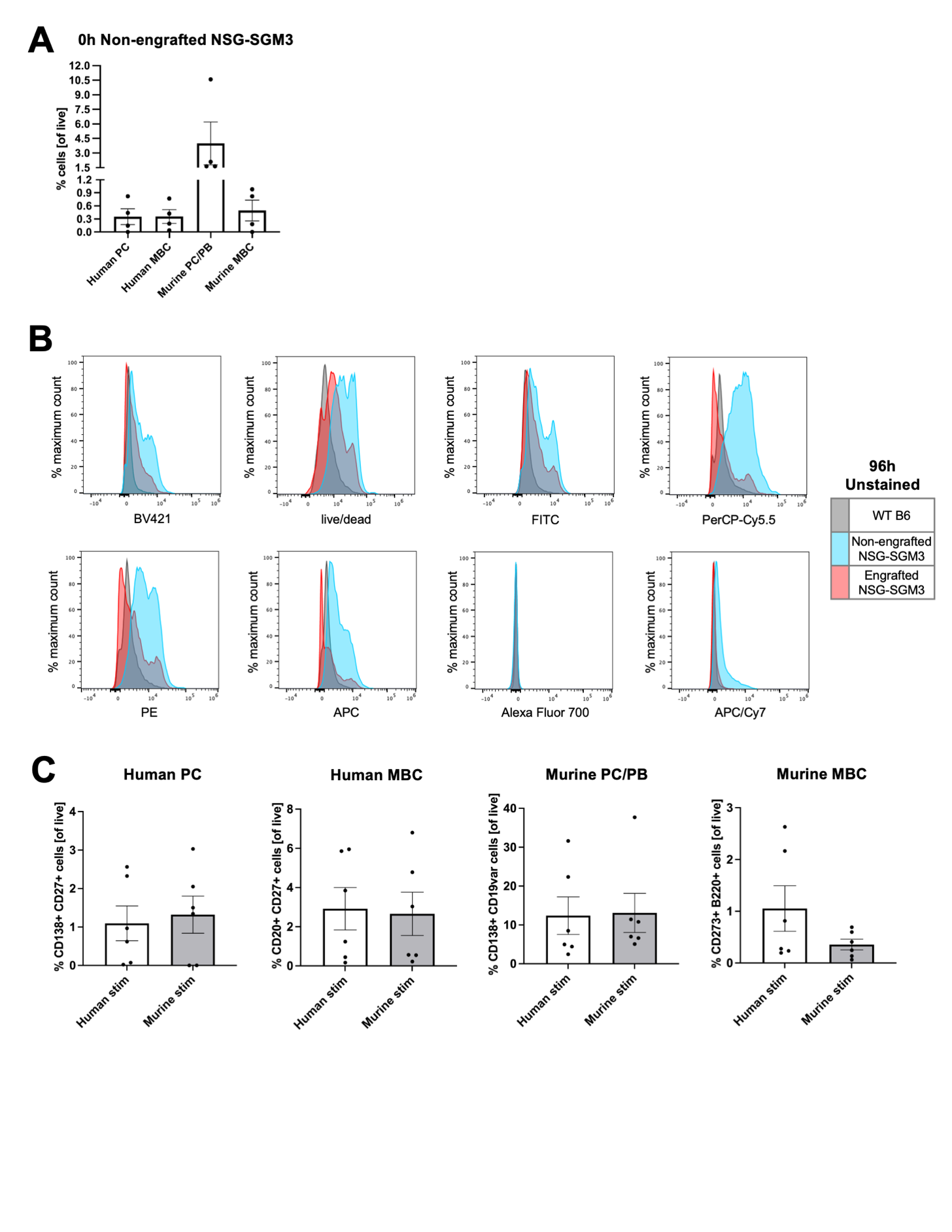

Supplement: Supplementary file 1 [file DataSheet1.docx]
